# Remarkable active-site dependent H_2_O promoting effect in CO oxidation

**DOI:** 10.1038/s41467-019-11871-w

**Published:** 2019-08-23

**Authors:** Shu Zhao, Fang Chen, Sibin Duan, Bin Shao, Tianbo Li, Hailian Tang, Qingquan Lin, Junying Zhang, Lin Li, Jiahui Huang, Nicolas Bion, Wei Liu, Hui Sun, Ai-Qin Wang, Masatake Haruta, Botao Qiao, Jun Li, Jingyue Liu, Tao Zhang

**Affiliations:** 1 Department of Chemistry and Key Laboratory of Organic Optoelectronics & Molecular Engineering of the Ministry of Education, Tsinghua University, 100084 Beijing, China; 2 Beijing Guyue New Materials Research Institute, Beijing University of Technology, 100124 Beijing, China; 3 CAS Key Laboratory of Science and Technology on Applied Catalysis, Dalian Institute of Chemical Physics, Chinese Academy of Sciences, 116023 Dalian, China; 4 Department of Physics, Arizona State University, Tempe, AZ 85287 United States; 5 Gold Catalysis Research Center, Dalian Institute of Chemical Physics, Chinese Academy of Sciences, 116023 Dalian, China; 6 Institute of Applied Catalysis, School of Chemistry and Chemical Engineering, Yantai University, 264005 Yantai, Shandong China; 7 Institut de Chimie des Milieux et Matériaux de Poitiers (IC2MP), University of Poitiers, CNRS, 4 rue Michel Brunet, TSA51106, F86073 Poitiers Cedex 9, France; 8 Dalian National Laboratory for Clean Energy, Dalian Institute of Chemical Physics, Chinese Academy of Sciences, 457 Zhongshan Road, 116023 Dalian, China; 9 Research Center for Gold Chemistry and Department of Applied Chemistry, Graduate School of Urban Environmental Sciences, Tokyo Metropolitan University, Tokyo, 192-0397 Japan; 10 Department of Chemistry, Southern University of Science and Technology, 518055 Shenzhen, China

**Keywords:** Catalytic mechanisms, Heterogeneous catalysis, Density functional theory

## Abstract

The interfacial sites of supported metal catalysts are often critical in determining their performance. Single-atom catalysts (SACs), with every atom contacted to the support, can maximize the number of interfacial sites. However, it is still an open question whether the single-atom sites possess similar catalytic properties to those of the interfacial sites of nanocatalysts. Herein, we report an active-site dependent catalytic performance on supported gold single atoms and nanoparticles (NPs), where CO oxidation on the single-atom sites is dramatically promoted by the presence of H_2_O whereas on NPs’ interfacial sites the promoting effect is much weaker. The remarkable H_2_O promoting effect makes the Au SAC two orders of magnitude more active than the commercial three-way catalyst. Theoretical studies reveal that the dramatic promoting effect of water on SACs originates from their unique local atomic structure and electronic properties that facilitate an efficient reaction channel of CO + OH.

## Abstract (web summary)

The issue that whether single-atom sites possess similar catalytic properties to the interfacial sites of nanocatalysts remains unresolved. Here, the authors demonstrate a large H_2_O promotional effect on CO oxidation over Au single-atom sites due to their unique local atomic structure and electronic properties.

## Introduction

Supported metal catalysts have played a central role in the modern chemical industry. The synergy between a support (typically a metal oxide) and supported phases (typically metal nanoparticles (NPs)) is often critical in determining the performance of catalysts because the interfacial perimeter sites in many cases serve as the dominant active sites^1–4^. Engineering the metal-support interfacial sites has therefore been a desirable approach to tailor the catalytic performance^5,6^. Heterogeneous single-atom catalysts (SACs)^7^, consisting of isolated metal single atoms (SAs) dispersed onto a support, maximize the number of interfacial perimeter sites^8,9^ and are expected to provide unique opportunities for catalyzing chemical transformations^10–12^. However, due to the quantum size and ensemble effects of metal clusters and particles, and the differences between SACs and nanocatalysts in electronic and support effects as well as metal-support interaction, it is still an open question whether the active sites in SACs possess similar catalytic properties to those of the interfacial sites in supported metal NPs or cluster catalysts.

Catalytic oxidation of CO has been one of the key heterogeneous catalytic processes that are essential to the significantly improved quality of life of our modern society^13^. Many experimental results have demonstrated that on reducible oxides supported metal catalysts, CO oxidation often occurs at the interfacial perimeter sites^1,2,14,15^, and the activity can be enhanced by the presence of interfacial OH-groups^6,16^ which are continuously supplied by the presence of water (H_2_O) in the reactant gas mixture^6,17–19^. Utilizing the residual H_2_O in the reaction system to enhance reactivity is of fundamental interest and practical importance, thus emerging as a frontier research topic^6,20^.

Herein we report a remarkable active-site dependent promoting effect of H_2_O on CO oxidation. We find that CO oxidation can be promoted by more than two orders of magnitude on CeO_2_ supported single Au atoms, while such a promoting effect is relatively small (less than 2 folds) on CeO_2_ supported Au NPs. Such a huge H_2_O promoting effect makes Au_1_/CeO_2_ SAC the most active catalyst for CO oxidation reported so far, and thus promises potential industrial applications. Computational modeling based on density functional theory (DFT) reveals that the significant difference in the water promoting effect originates from the different active-site structure and electronic properties between Au single atoms in the Au SAC and the interfacial Au atoms in supported Au NP catalysts. The positively charged, non-zero valent Au atom in the Au_1_/CeO_2_ SAC is more variable in oxidation states (e.g., Au(III), (II), (I)) as an electron acceptor, which offers a more efficient channel for the CO + OH reaction pathway with the presence of OH-groups (or water molecules). In contrast, the near zero-valent Au interfacial atoms in supported Au NPs do not seem to favor such oxidation state change, thus significantly hindering the CO + OH reaction pathway.

## Results

### Preparation and characterization of catalysts

CeO_2_ supported Au SACs (denoted as Au_1_/CeO_2_) were prepared by a previously developed electrostatic adsorption method^9,21,22^. A very low Au loading of 0.03 wt% was used to ensure all Au atoms are isolated and remain as SACs^21^. In addition, this loading is comparable to the level of commercial three-way catalysts (TWCs)^23^, thus being convenient to perform catalytic test comparison with those of TWCs. A 0.98 wt% Au/CeO_2_ reference catalyst (denoted as Au/CeO_2_-RRCe, provided by Haruta Gold Inc.) and a 0.03 wt% Au/CeO_2_ nanocatalyst consisting of only colloidal Au NPs (denoted as Au/CeO_2_-NP) were also studied for comparison. Details on materials and sample preparation procedures are presented in Methods section. Aberration-corrected high-angle annular dark-field scanning transmission electron microscopy (ac-HAADF-STEM) provides unambiguous information on the dispersion of supported metal catalysts, especially SACs^24^. Representative ac-HAADF-STEM images with various magnifications presented in Supplementary Figs. 1 and 2 reveal clearly that only isolated individual Au atoms were detected in the Au_1_/CeO_2_ SAC, while only Au NPs with sizes of 2–4 nm were observed in the Au/CeO_2_-NP (the low number density of the Au NPs is due to the very low loading level). The average size of the Au NPs in the Au/CeO_2_-RRCe catalyst was estimated to be about 5 nm in diameter (provided by the supplier).

### Activity test of Au_1_/CeO_2_ SAC on CO oxidation

**Fig. 1:**
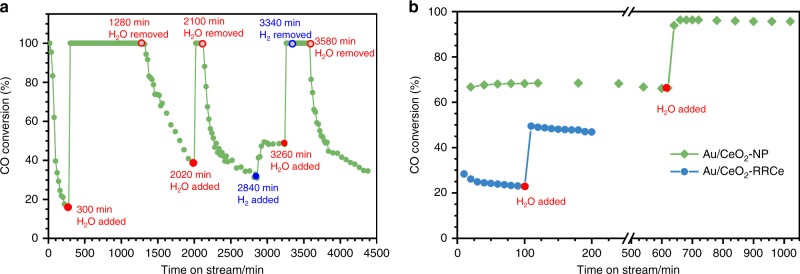
CO conversion rate as a function of reaction time for CO oxidation at 200 °C. **a** 0.03 wt% Au_1_/CeO_2_, and **b** Au/CeO_2_-NP and Au/CeO_2_-RRCe. Reaction condition: 1 vol% CO +1 vol% O_2_ He balance with a flowrate of 33.3 mL min^−1^; 20 mg catalyst was used. The added H_2_O amount was about 2 vol% while H_2_ was about 1 vol%

For CO oxidation measurements, a gas mixture consisting of 1 vol% CO + 1 vol% O_2_ and helium balance was used. Supplementary Fig. 3 shows that the Au_1_/CeO_2_ SAC exhibited extremely high activity for CO oxidation (Run 1 in Supplementary Fig. 3a), which is only slightly lower than that of the Au/CeO_2_-RR2Ce with a much higher Au loading of 0.98 wt% (Run 1 in Supplementary Fig. 3d). However, during the second run (Run 2 in Supplementary Fig. 3a) the activity decreased dramatically. Previous studies have suggested that supported Au catalysts generally deactivate due to sintering of the Au species (dominant at high temperature) and/or the buildup of carbonates on the active sites (prominent at low temperature). In order to better understand the origin of the dramatic deactivation in the second run on the Au_1_/CeO_2_ SAC, we conducted a stability test at 200 °C, the highest reaction temperature in our cycle tests. Unexpectedly, the CO conversion rate dropped rapidly from 100% to <20% within 300 min (Fig. 1a). We hypothesized that the buildup of carbonates should not play a major role in the observed deactivation because the reaction temperature is relatively high. The fact that a helium and oxygen treatment at 200 °C did not recover the deactivated catalyst further confirmed this hypothesis (Supplementary Fig. 4)^7^. We also believe that sintering of Au_1_ atoms should not be responsible for the observed deactivation because of the following: (1) the loading level of the Au_1_ is extremely low, and (2) no sintering of Au_1_ atoms after long-term reaction was observed in our previous work^21,22^. Furthermore, ac-HAADF-STEM images of the deactivated catalyst did not show any Au nanoclusters or NPs (Supplementary Fig. 5), directly confirming that the deactivated catalyst contains only isolated Au_1_ atoms. The observed fast deactivation is therefore proposed to stem from the consumption of OH species^18,25,26^. To test this hypothesis, we spiked ~2 vol% water into the reaction gas mixture after the Au_1_/CeO_2_ SAC had deactivated. As clearly shown in Fig. 1a, full CO conversion was immediately restored. With the presence of H_2_O, the Au_1_/CeO_2_ SAC ran stably for at least 1000 min with complete CO conversion. When H_2_O was removed from the reaction gas mixture the CO conversion dropped immediately. Such an activity modulation of CO oxidation by H_2_O was repeated for several cycles, confirming that the presence of OH/H_2_O plays a dominant role in controlling the activity of CO oxidation on the Au_1_/CeO_2_ SAC. Besides, an operando diffuse reflectance Fourier transform infrared (DRIFT) study reveals that carbonate species indeed formed during CO oxidation without the presence of H_2_O. The addition of H_2_O, however, did not change the nature and amount of such deposited carbonate species (Supplementary Fig. 6), verifying that the presence of the carbonate species is not responsible for the experimentally observed rapid deactivation and activity recovery. We also tested the effects of adding H_2_ to the reactants and the result showed that the addition of 1 vol% H_2_ promoted the CO oxidation as well, primarily due to the fact that oxidation of H_2_ formed H_2_O, which could subsequently promote the CO oxidation^21^.

By simply examining Supplementary Fig. 3a, the deactivation during Run 1 was not so obvious. At least two factors contribute to this: (i) the catalyst should deactivate slower at lower temperatures than at 200 °C because the loss rate of the OH groups is slower and (ii) the conversion rate increases with reaction temperature, which offsets the deactivation with reaction time. Therefore, from the Run 1 plot (conversion vs. reaction temperature) in Supplementary Fig. 3a one cannot easily detect the rate of deactivation of the evaluated catalyst. To verify our hypothesis, we further conducted a stability test at 160 °C. As clearly shown in Supplementary Fig. 7, the Au_1_/CeO_2_ SAC certainly deactivated but the deactivation rate was much slower than that at 200 °C. The introduction of water, again, immediately recovered the activity, similar to the behavior at 200 °C.

The CO conversion rates as a function of reaction temperature were also measured in the presence of H_2_O in order to further understand the effects of adding H_2_O on the activity of the Au_1_/CeO_2_ SACs. As clearly shown in Supplementary Fig. 3b, for the first run the addition of H_2_O improved the activity obviously, which shifted the total CO conversion temperature from 120 to 80 °C. Of more importance, the activity in the second run (Run 2) did not decrease at all but slightly increased, confirming that the presence of H_2_O maintained the catalyst activity. The slight activity increase in the Run 2 may originate from the catalyst modification by the reaction gas^27^ and/or the H_2_O vapor during the first run. Especially the latter can have significant promoting effect on the catalytic performance of SACs^28^. In addition, since our Au_1_/CeO_2_ SAC was dried at 60 °C without further calcination at elevated temperatures, a subtle change in catalyst (surface) structure as well as metal-support interaction between Au atoms and CeO_2_ surfaces may occur during the first run.

### Effects of H_2_O on Au SAC and nanocatalysts

The effects of H_2_O on CO oxidation over supported Au NPs have been extensively studied^17,29–31^. Especially, for CeO_2_-supported Au catalysts the presence of water has only slight^32^ or negligible^33^ promotional effect. To investigate the promoting effect of H_2_O on CeO_2_-supported Au NP catalysts, we further tested the Au/CeO_2_ NP and the Au/CeO_2_-RRCe catalysts under the same reaction condition with and without the presence of water. Figure 1b demonstrates that the addition of H_2_O promotes CO oxidation even on CeO_2_-supported Au NP catalysts; the promotional effect is, however, much smaller compared to that on the Au_1_/CeO_2_ SAC. Supplementary Fig. 3c, d show a much smaller H_2_O promoting effect in the cycling tests as well, further confirming the weak promoting effect of H_2_O on CeO_2_ supported Au nanocatalysts.

A quantitative comparison of the H_2_O promotional effect was further performed by measuring the specific reaction rates of CO oxidation with or without the presence of H_2_O on various catalysts (Supplementary Table 1). The reference Au/CeO_2_-RR2Ce was first tested to benchmark our catalytic measurement system. At ambient temperatures (27 °C), Au/CeO_2_-RR2Ce catalyst had a specific rate of 0.13 mol_CO_ h^−1^ g_Au_^−1^ (Entry 1 in Supplementary Table 1), yielding a turnover frequency (TOF) of 0.036 s^−1^, similar to that of the previously reported CeO_2_-supported Au catalysts^34,35^, suggesting the test system is robust. The CO conversion on both the Au_1_/CeO_2_ and Au/CeO_2_-NP catalysts were undetectable at ambient temperatures, probably due to the extremely low level of Au loading (0.03 wt%) in these catalysts (Entry 2 and 5). When ~2 vol% water was added, the CO conversion on the Au/CeO_2_-NP was still undetectable, suggesting a weak, if any, promoting effect. With the addition of ~2 vol% water, however, the Au_1_/CeO_2_ yielded a specific rate of 3.6 mol_CO_ h^−1^ g_Au_^−1^ (TOF of 0.2 s^−1^), about 30 times higher than that of Au/CeO_2_-RR2Ce. The specific rate is also double that of the Au/TiO_2_-RR2Ti (1.53 mol_CO_ h^−1^ g_Au_^−1^), one of the most active catalysts for CO oxidation^23^, suggesting the extremely high activity of the Au_1_/CeO_2_ SAC in the presence of H_2_O. At higher reaction temperatures the addition of H_2_O decreased the specific rates on the Au/CeO_2_-NP slightly at 100 °C and increased it by only 0.6 times at 200 °C (Entry 6, 7); whereas on Au/CeO_2_-RRCe about 1.9 times increment was observed at 200 °C (Entry 8). On the other hand, on the Au_1_/CeO_2_ SAC the specific rates increased more than 190 and 150 times at 100 and 200 °C, respectively (Entry 3, 4). These results unambiguously demonstrate that the H_2_O promotional effect on the CeO_2_ supported Au_1_ atoms is significantly larger than that on the CeO_2_-supported Au NP catalysts.

**Fig. 2:**
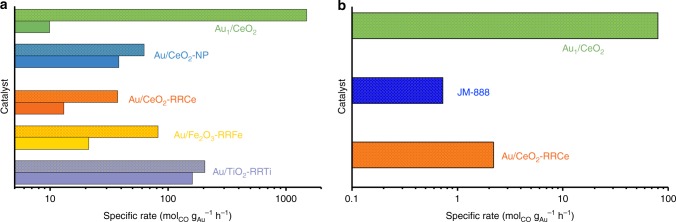
Comparison of CO oxidation activities on different catalysts. **a** Specific rate of CO oxidation at 200 °C with (patterned bar) or without (solid bar) the presence of H_2_O on Au_1_/CeO_2_, Au/CeO_2_-NP and various standard Au catalysts provided by Haruta Gold Inc. Reaction gas: 1 vol% CO +1 vol% O_2_ (+2 vol% H_2_O) balanced with He; **b** Specific rate of CO oxidation under simulated practical conditions on Au_1_/CeO_2_, Au/CeO_2_-RRCe and commercial JM-888. Reaction gas composition: 1.6 vol% CO, 1 vol% O_2_, 0.01 vol% propene, 0.0087 vol% toluene, 10 vol% water and balanced with He

To confirm the unique H_2_O effect on the Au_1_/CeO_2_ SAC we further evaluated the H_2_O promotional effect on several other standard Au catalysts provided by Haruta Gold Inc. Compared to that on the Au_1_/CeO_2_ SAC the promoting effect of H_2_O on CO oxidation on all these supported Au catalysts is small (Entry 9–10, Supplementary Table 1). It should be noted that Supplementary Fig. 8 shows that the CeO_2_ itself is neither active for CO oxidation nor promoted by the presence of H_2_O, suggesting that the unique promotional effect originated from the presence of Au_1_ single atoms and/or the synergistic effects between CeO_2_ surfaces and Au_1_ single atoms. Furthermore, the catalytic test data suggest that with the addition of H_2_O our Au_1_/CeO_2_ SAC yielded a specific reaction rate as high as 1500 mol_CO_ h^−1^ g_Au_^−1^ at 200 °C (Fig. 2 and Supplementary Table 1), about 7–40 times higher than that of various standard Au catalysts. To the best of our knowledge, this is the highest specific reaction rate for CO oxidation at similar temperatures ever reported in literature.

Since our Au_1_/CeO_2_ SAC exhibited such high activity with the presence of H_2_O, we further evaluated the practical utility of such Au_1_/CeO_2_ SACs. Supplementary Fig. 9 shows the activity of an Au_1_/CeO_2_ SAC with extremely low levels of Au_1_ loading (0.005 wt%, i.e., 50 ppm). This catalyst totally converted CO to CO_2_ at 150 °C, meeting the requirement of the so-called 150 °C challenge^23,36^. In the second cycle test (Run 2 in Supplementary Fig. 9) the activity did not change appreciably, suggesting excellent stability with the presence of H_2_O during CO oxidation.

### Application in simulated CO emission control

Previous studies demonstrated that supported Au catalysts possess higher activity for CO oxidation at ambient temperatures than that of supported PGM catalysts but they are usually less active at elevated temperatures (>200 °C)^37^. In order to understand the catalytic properties of our Au_1_/CeO_2_ SACs for practical applications, we evaluated the performance of our Au_1_/CeO_2_ sample and a commercial three-way catalyst (TWC) supplied by Johnson Matthey (JM-888) in a simulated CO emission control test^23^. Supplementary Fig. 10 clearly shows that even with 10 times higher metal space velocity our Au_1_/CeO_2_ still exhibited a much higher conversion rate than that of the commercial JM-888 catalyst: The temperature of CO total conversion is more than 100 °C lower than that on the JM-888. The long-term stability tests on both catalysts at 200 °C under the simulated CO emission control condition are shown in Supplementary Fig. 11. Both catalysts exhibited a similar stability performance, i.e., after a fast deactivation at the initial stage of the CO oxidation both catalysts were stable during the near 70-h test. Such a stability behavior of the Au_1_/CeO_2_ SAC is similar to that during the PROX reaction^21^, further confirming the good stability of the single Au atoms supported on CeO_2_. Moreover, we estimated that our Au_1_/CeO_2_ SAC (a specific reaction rate of 80 mol_CO_ g_Au_^−1^ h^−1^ at 200 °C) is ~100 times more active than that of the commercial JM-888 (a specific reaction rate of 0.73 mol_CO_ g_Pt+Pd_^−1^ h^−1^). The reference catalyst Au/CeO_2_-RRCe exhibits an activity (a specific reaction rate of 2.2 mol_CO_ g_Au_^−1^ h) that is only slightly higher than that of the commercial JM-888 under identical test conditions. Both the extremely high activity and excellent stability of the Au_1_/CeO_2_ SACs suggest tremendous potential and advantages over the current commercial catalysts (e.g., significant cost savings) for practical applications.

### DFT studies

DFT calculations were carried out to understand the active-site geometry dependent H_2_O promoting effect and the fundamental processes that differentiate Au_1_/CeO_2_ SAC from those of the CeO_2_-supported Au NP catalysts. Inasmuch as the CeO_2_ was synthesized by a co-precipitation method and existed in a small nanoparticle form, no preferential surface of the CeO_2_ was exposed. We adopted the model structure with Au single atoms doped on CeO_2_ (111) surface, which is the most stable surface of ceria and Au single atoms can be rather stable on such surface^38^. Three models were used to represent the Au_1_/CeO_2_ (111) catalyst (see Supplementary Fig. 12 for details). Two reaction pathways were investigated to explore the catalytic cycle in the absence (pathway A) or the presence (pathway B) of OH-group taking part in the CO oxidation reaction.

Generally the CO oxidation on ceria-supported metal NP catalyst is governed by a Mars-van-Krevelen (MvK) mechanism^26,28,39^. With model A (Supplementary Fig. 12a) the energy for O vacancy (O_v_) formation is as low as 0.13 eV without any reaction barrier (Supplementary Table 2)^39^, resulting in ultrahigh activity. However, the calculated result with such a model does not fit well with our experimental data and the reported results, indicating that the bare ceria surface with a number of dangling bonds is too simplistic^40,41^. Considering that in realistic reaction condition CeO_2_ usually contains ubiquitous H_2_O molecules or water decomposition induced surface OH-group, which can partly be removed only by ultra vacuum or high-temperature treatment (>800 °C)^42^, we propose that model B where the CeO_2_ surface is hydroxylated except one of the three surface O atoms directly bonded to Au_1_ atom is more appropriate to compare with experimental condition, as shown in previous work^43^. The calculated O_v_ formation energy (1.42 eV, Supplementary Table 2) becomes much higher and the whole reaction needs to overcome a barrier of about 0.55 eV (details were presented in Supplementary Fig. 13). As a result, the presence of the hydroxyl species on the ceria surfaces suppresses the MvK process both thermodynamically and kinetically.

**Fig. 3:**
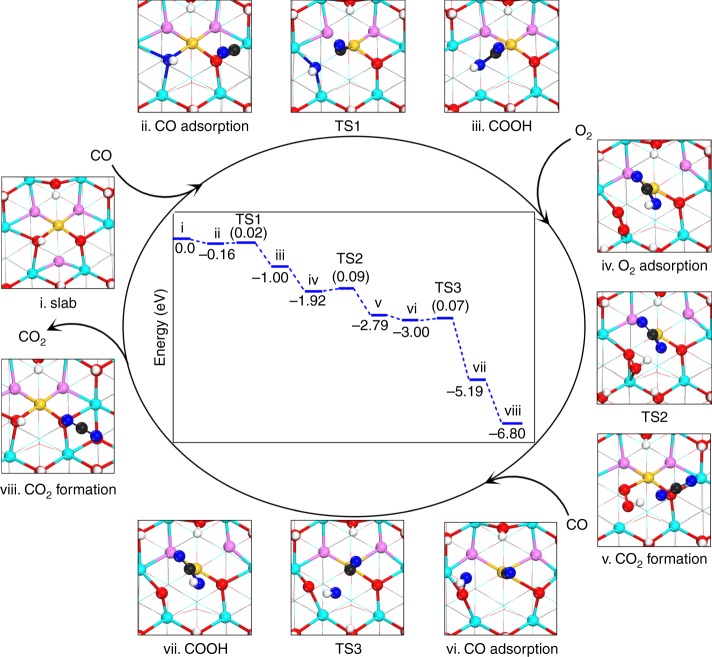
Reaction pathway B for CO oxidation on the Au_1_/CeO_2_ (111). The inset (within the ellipse) shows the potential energy profile and the numbers in the parentheses indicate the barriers (in eV) of elementary steps

With the presence of OH groups on the CeO_2_ surface, we investigated another reaction channel CO + OH → COOH → CO_2_ + H (pathway B). The calculated energy profile and detailed structures are presented in Fig. 3 and Supplementary Fig. 14, respectively. The catalytic cycle has a barrier as low as 0.09 eV, much more favorable than the MvK process. The probable reaction of COOH^*^ + OH^*^ → CO_2_ + H_2_O (Supplementary Fig. 15) was also calculated and this process needs to overcome a barrier of 0.24 eV and exothermic by −1.00 eV. The low energy barrier indicates that the OH-groups can be consumed easily by forming H_2_O during reaction. This is in good agreement with the experimental result that CO conversion on the Au_1_/CeO_2_ SAC dropped rapidly without the continuous supply of H_2_O. After the OH species (adjacent to the Au_1_ site) were consumed, as indicated in model C (Supplementary Fig. 12c) where the CeO_2_ surface is hydroxylated except the three surface O atoms bonded to Au_1_ atom, the O_v_ formation energy becomes very high (1.23 eV, Supplementary Table 2), suggesting that the presence of nearby OH species on the CeO_2_ surface, which may not directly connected to the Au_1_ atom, can also significantly suppress the MvK process. This calculation clearly elucidates that the deactivation of the Au_1_/CeO_2_ SAC is determined by the availability of the OH species to the anchored Au_1_ atoms. During the CO oxidation process, even if the OH species directly bonded to the Au_1_ atoms are consumed the nearby OH species on the CeO_2_ surface can still significantly hinder the MvK process.

Water dissociation on ceria is facile with a low energy barrier of 0.29 eV and exothermic reaction energy of −0.72 eV (Supplementary Fig. 15), resulting in plenty of surface hydroxyls that can replenish the consumed OH-groups. As presented in Supplementary Table 3, the calculated rate constant of pathway B is several orders of magnitude higher than that of pathway A, which is consistent with the experimental results. These DFT calculations confirm that the surface OH-groups directly participate in the CO oxidation reaction rather than only serving as bystander species. In addition, the proton transfer from COOH^*^ to O_2_^δ-^ and OH^*^ has similar rate constant, and thus surface OH-groups can be consumed through COOH reaction with OH^*^ to form H_2_O (Supplementary Table 3). Furthermore, the role of O_2_ in pathway B is mainly to help decomposing COOH into CO_2_ and H which is different from that of pathway A where the role of O_2_ is primarily to replenish the consumed O.

**Fig. 4:**
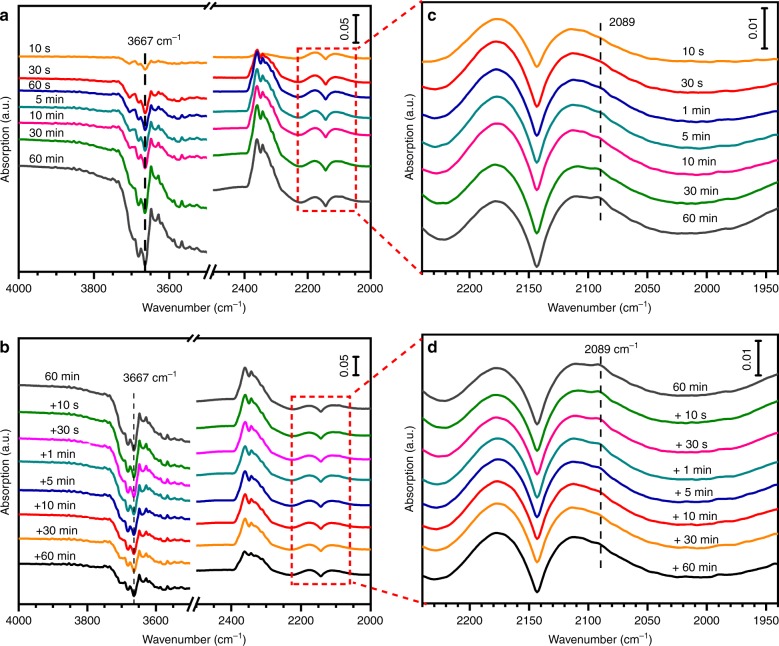
In-situ DRIFT spectra of Au_1_/CeO_2_ as a function of reaction time at 200 °C. **a**: CO oxidation without the presence of H_2_O. Reaction condition: 1 vol% CO +2 vol% O_2_ balanced He, flowrate: 50 mL min^−1^; and **b**: introducing 2 vol% H_2_O after **a**. Here **c** and **d** are enlargement of the corresponding spectra in box in **a** and **b**, respectively

An operando DRIFT study of the Au_1_/CeO_2_ under CO oxidation with or without the presence of H_2_O (Fig. 4) provides experimental evidence to support the DFT calculations. As shown in Fig. 4a, without the presence of H_2_O the peak of the structural OH-group (~3670 cm^−1^)^42^ decreases gradually accompanied by the appearance and the gradual increase in the strength of a 2089 cm^−1^ band. Considering the extremely low loading of Au (probably below the detection limit of DRIFT) and the positively charged chemical state of the Au atoms in the Au_1_/CeO_2_ SAC, it is highly reasonable to ascribe this band to CO adsorption on Ce^3+^ rather than on Au_1_. The reported data suggest that the band for CO adsorption on Ce^3+^ usually locates in the range of 2120–2150 cm^−1,^^44,45^. In this particular case, we believe that the hydroxylation of the CeO_2_ surfaces may have shifted the band position slightly. We performed a DFT calculation of CO adsorption on the hydroxylated CeO_2_ surface and found that the adsorption band lies at a frequency of 2072 cm^−1^, close to our experimentally observed values (Fig. 4a). Therefore, we tentatively ascribed this band at 2089 cm^−1^ to CO adsorption on Ce^3+^ on hydroxylated CeO_2_ surfaces. Therefore, in Fig. 4a the peak decrease of the OH-group suggests a gradual consumption of OH species while the appearance of the band due to CO adsorption on Ce^3+^ evidences the reduction of Ce^4+^ during the CO oxidation process without the continuous supply of H_2_O. The lattice O participated in the reaction and the CeO_2_ was partially reduced after the OH-groups were consumed. After the introduction of H_2_O, as shown in Fig. 4b, the structural OH-group was gradually compensated and the CO adsorption on Ce^3+^ gradually disappeared. This set of operando investigation unambiguously demonstrates that the MvK mechanism dominates without a continuous supply of H_2_O to the CeO_2_ surfaces, while such an MvK process does not play a major role when H_2_O molecules are continuously supplied to the CeO_2_ surfaces.

The promoting effect of the OH-group on the activity of CO oxidation on Au/CeO_2_-NP catalyst was also calculated with a model of Au_13_/CeO_2_ (111)^46,47^. As shown in Supplementary Fig. 16 and Supplementary Table 2, the adsorption energy of CO is independent of the presence of OH species on the CeO_2_ surface. The reaction energy for CO + O_L_ → CO_2_ + O_V_ is similar to that of the Au_1_/CeO_2_ SAC (−1.00 vs. −0.96 eV). In addition, CO reaction with surface OH to form COOH* on Au/CeO_2_-NP is endothermic by 0.67 eV (Supplementary Table 2), which is thermodynamically unfavorable. Thus, we can deduce that water has a weak promoting effect on the activity of Au/CeO_2_-NP, which is consistent with the experimental results.

Based on the computational modeling we can conclude that the most important difference between single-atom sites in SACs and interfacial sites in NP catalysts is the different environments of Au atoms that correspondingly result in different electronic properties (Supplementary Table 2). In SACs, the Au_1_ atoms are located at the Ce vacancy sites: Each Au_1_ atom bonds to three coordinately unsaturated O atoms resulting in highly positively charged state (with a calculated Bader charge of +1.10 eV) due to electron transfer. In supported Au NP catalysts, the Au atoms located at the interfacial sites are bonded to only one coordinately saturated O atom and have little electron transfer to the support O atom, thus existing in almost metallic state (with Bader charge of +0.03 eV). The presence of the highly positively charged Au_1_ single atoms is crucial to the CO + OH reaction channel due to their flexibility in variation of oxidation state; the Au_1_ atom can be reduced from Au(III) to Au(II) or Au(I), with Bader charge decreased from +1.10 to +0.69 eV during the CO oxidation reaction (Supplementary Fig. 17 and Supplementary Table 2), which makes the reaction step exothermic. On the other hand, the primarily metallic state of the Au interfacial atoms makes the oxidation state change more difficult, if not impossible, resulting in an endothermic reaction. The recent finding that gold single atoms can be dynamically formed during CO oxidation reaction seem to provide a connection to covert metallic gold atoms of the NPs to non-zero-valent gold single atoms under certain conditions^40,48^.

In summary, a remarkable active-site-dependent H_2_O promoting effect in CO oxidation has been discovered and investigated experimentally and computationally. The catalytic nature of the single Au atoms supported on CeO_2_ is dramatically sensitive to the presence of H_2_O, which renders the Au_1_/CeO_2_ SAC extremely active for CO oxidation. On the other hand, the perimeter Au atoms in CeO_2_ supported Au NP catalysts do not possess such unique properties. DFT calculations illustrated the intrinsic differences between the Au_1_ single-atom sites in SACs and the interfacial perimeter Au sites in supported Au NP catalysts. The differences in the environment between the Au_1_ atoms and the perimeter Au atoms are responsible for the experimentally observed huge differences in CO oxidation activity when H_2_O is in the reactant gas mixture. The gold single-atom catalyst is two orders of magnitude more active than the commercial three-way catalyst, which holds promises for future practical applications of single-atom catalysts. This work provides an avenue to develop highly efficient catalyst for CO oxidation with the presence of water by fabrication of suitable single-atom catalysts.

## Methods

### Catalyst preparation

CeO_2_ support was prepared by a co-precipitation method^9,21^. In details, an aqueous solution of cerium nitrate hexahydrate (Ce(NO_3_)_3_·6H_2_O, 1 mol L^−1^) was added dropwise to an aqueous solution of sodium carbonate (Na_2_CO_3_, 1 mol L^−1^) under vigorous stirring (1500 r.p.m.) at 50 °C, with the pH value of the resulting solution controlled at *ca*. 8. After continuing stirring at the same speed (1500 r.p.m.) and aging for 3 h, respectively, the resulting precipitate was recovered by filtration and multiple washing. The recovered solid was dried at 60 °C for 5 h in oven and then calcined at 400 °C for 5 h in a muffle oven at a heating rate of 10 °C min^−1^ from room temperature.

CeO_2_ supported Au SACs (denoted as Au_1_/CeO_2_) were prepared by a facile electrostatic adsorption method^9,21^. Typically, 1 g CeO_2_ powder was dispersed in deionized water with stirring (800 r.p.m.). Appropriate amount of HAuCl_4_ (AuCl_3_ dissolved in diluted hydrochloric acid, corresponding to an Au loading of 0.03 wt%) solution was added dropwise into the CeO_2_ suspension solution under stirring at room temperature. After continuous stirring (600 r.p.m.) for 2 h and followed by aging for 2 h, the solution was filtered and washed with deionized water for several times, and then dried at 60 °C for 5 h in an oven without any further heat treatment.

CeO_2_-supported Au NP catalysts were prepared via a colloidal deposition method by using Poly(vinyl alcohol) (PVA, Mw10,000 from Aldrich, 80% hydrolyzed) as protecting agent^22^ with a Au loading of 0.03 wt% (denoted as Au/CeO_2_-NP). In a typical procedure, 0.3 mg of HAuCl_4_ solution (AuCl_3_ dissolved in diluted hydrochloric acid) and 0.2 mL of 1 mg mL^−1^ PVA solution (Au: PVA = 1.5: 1 mg mg^−1^) were added to 50 mL of deionized water under vigorous stirring. After 10 min, 2 mL of 0.004 mol L^−1^ NaBH_4_ solution (Au: NaBH_4_ = 1: 5 mol mol^−1^) was rapidly injected to the solution to obtain a dark orange-brown solution, indicating the formation of gold colloid. One gram of CeO_2_ powder was then added immediately and after 8 h the gold colloid was completely adsorbed. It should be noted that to avoid contact with light all containers were covered with aluminum foil during the synthesis process. The solids were collected by filtration and washed for several times with deionized water.

A CeO_2_ supported Au nanocatalyst (with 0.98 wt% Au loading) provided by Haruta Gold Inc. was also used as a reference catalyst (denoted as Au/CeO_2_-RRCe), which consists of mainly Au NPs with an average diameter of 4.5 nm and were prepared by a deposition-precipitation method and calcined at 400 °C.

### Catalyst characterization

The loading of Au was determined by inductively coupled plasma atomic emission spectroscopy (ICPAES) using an IRIS Intrepid II XSP instrument (Thermo Electron Corporation).

Sub-angstrom-resolution high-angle annular dark-field (HAADF) scanning transmission electron microscopy (STEM) images were obtained on the JEOL JEM-ARM200F TEM/STEM with a guaranteed resolution of 0.08 nm. Before microscopy examination, the samples were ultrasonically dispersed in ethanol and then a drop of the solution was deposited onto a copper grid coated with a thin lacey carbon film.

### Catalytic performance test

The catalytic performances of the catalysts for CO oxidation with or without the presence of H_2_O were evaluated in a fixed-bed reactor. Eighty milligram of the sample was loaded in a straight quartz reactor. After purged with He for 10 min, the feed gas containing 1 vol% CO, 1 vol% O_2_ and balance He was allowed to pass through the reactor at a flowrate of 33.3 mL min^−1^ or higher, corresponding to a weight hourly space velocity (WHSV) of ~25,000 mL·h^−1^ g^−1^_cat_ or higher (the exact WHSV is noted in the text discussions). For reaction with the presence of H_2_O the feed gas is 1 vol% CO, 1 vol% O_2_, 2 vol% H_2_O and balance He. The effluent gas compositions were on line analyzed by a gas chromatograph (HP 7890 A) equipped with an Agilent SC-ST 80/100 column and a thermal conductivity detector using He as carrier gas. The CO conversions were calculated based on the difference between inlet and outlet CO concentrations.

For measurements of specific reaction rates, CO oxidation reactions were conducted at a differential mode where the GHSV was up to ~3,125,000 ml g_cat_^−1^ h^−1^ and the CO conversions were kept below 30 %. Towards this goal, 10–80 mg of the sample with a size of 200 mesh was diluted with ~80 mg Al_2_O_3_ (size was also around 200 mesh). For each run at a specified reaction temperature, the CO conversions at 20, 40, and 60 min were averaged and used for calculations of the specific rate. The turnover frequency (TOF) was then calculated based on the specific rate and the dispersion where the dispersion of SACs was assumed to be 100% and the dispersion of the Au/CeO_2_ NP catalysts was calculated according to the equation D = 0.9/d where D represents the dispersion and d is the average diameter of the Au NPs (in nm).

### Theoretical and computational details

All of the theoretical calculations were performed using periodic density functional theory (DFT) as implemented in the Vienna ab initio simulation package (VASP)^49,50^. The electron exchange and correlation energy was treated within the generalized gradient approximation in the Perdew-Burke-Ernzerhof formalism (GGA-PBE)^51^. The electron-ionic interaction was described by the projector augmented wave (PAW) method^52,53^. Spin-polarized DFT + U calculations with a Hubbard correction value of U_eff_ = 5.0 eV for the Ce 4 *f* state were applied to correct the strong electron-correlation properties of CeO_2_^54,55.^ The valence orbitals of Ce *(*4 *f*, 5 *s*, 6 *s*, 5*p*, 5*d*), Au (5*d*, 6 *s*), C (2 *s*, 2*p*), O (2 *s*, 2*p*), and H (1 *s*) were described by plane-wave basis sets with cutoff energies of 400 eV, whereas the Brillouin zone was sampled at the Γ-point. The convergence criteria for the electronic self-consistent iteration and force were set to 10^–4^ eV and 0.03 eV/Å, respectively. For evaluating the energy barriers, all transition states were located using the climbing image nudged elastic band (CI-NEB) method^56,57^, and harmonic vibrational frequencies were analyzed to evaluate a transition state with only one imaginary frequency.

The CeO_2_ (111)-*p*(3 × 3) surface was used to model the CeO_2_ substrate, consisting of 3 O-Ce-O tri-layers (nine atomic layers), and only the bottom three atomic-layer were frozen while the remaining layers were allowed to relax. The slab was repeated periodically with a vacuum depth of around 15 Å in the direction of the surface normal. In our surface model, the Au_1_ atom is located at the substitutional position of the surface Ce atom, which is consistent with the experimental data^21^. Three models were used to simulate the structure of Au_1_/CeO_2_.

Model A: Au atoms substituted one of the Ce atom, same as the experimental image, Supplementary Fig. 12a^39^.

Model B: similar to A but all the surface oxygen atoms were hydroxylated except one of the three bonded to gold atoms. The optimized surface structure is presented in Supplementary Fig. 12b. The reason that we hydroxylated the surface oxygen is based on the following facts: (1) in real catalytic reaction, water is unavoidable so that the ceria surface can hardly remain as bare, unhydroxylated one with numerous dangling bonds. With presence of water, ceria surface can easily be hydroxylated; (2) Farnesi et al. found that Au^+^ on the bare CeO_2_(111) surface can catalyze the CO oxidation without barrier energy^39^. However, all experimental data, including our results here, reveal that dry Au_1_/CeO_2_ single-atom catalysts are not highly active^41,58^, implying non-negligible barrier for CO conversion. Considering that the CeO_2_ always contains a certain amount of hydroxyl group on surface of real catalysts^42^, we hydroxylated the surface to make the model more realistic.

Model C: similar to A but all the surface oxygen atoms were hydroxylated except the three bonded to gold atoms. This model was used to simulate the sample after OH-group being consumed during CO oxidation reaction.

The adsorption energies were calculated according to the equation, *E*_ads_ = *E*(adsorbate/slab) − [*E*(slab) + *E*(adsorbate)], in which *E*(adsorbate/slab), *E*(adsorbate), and *E*(slab) are the calculated energies of species adsorbed on the surface, a gaseous-phase molecule and the bare surface, respectively. The reaction energy and barrier were calculated by *E*_*r*_ = *E*(FS) − *E*(IS) and *E*_a_ = *E*(TS) − *E*(IS), where *E*(IS), *E*(FS) and *E*(TS) are the energies of the corresponding initial state (IS), final state (FS), and transition state (TS), respectively.

## Supplementary information

Supplementary information

Peer Review

## Data Availability

The data that support the findings of this study are available within the paper and its Supplementary Information, and all data are available from the authors on reasonable request.
