# Supplementary material for: Remarkable active-site dependent H2O promoting effect in CO oxidation
Source: Nat Commun. 2019 Aug 23;10:3824. doi: 10.1038/s41467-019-11871-w (PMC6707188; doi:10.1038/s41467-019-11871-w)
Supplement: Supplementary file 1 — Supplementary information [file 41467_2019_11871_MOESM1_ESM.pdf]

Remarkable active-site dependent H_2O promoting effect in CO oxidation

Zhao et al.

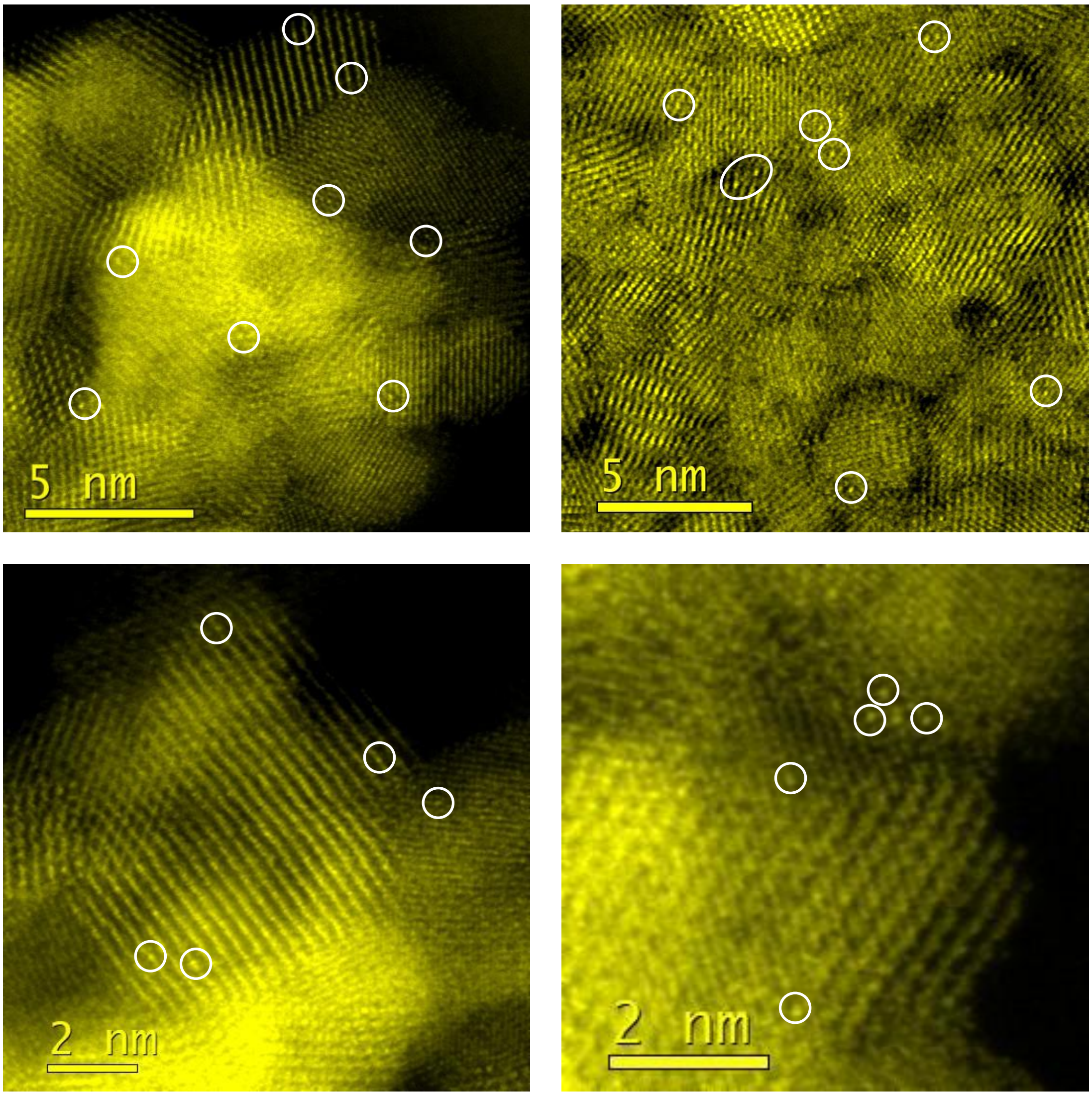

Supplementary Figure 1. Representative HAADF-STEM images of Au_1/CeO_2 with different magnifications.

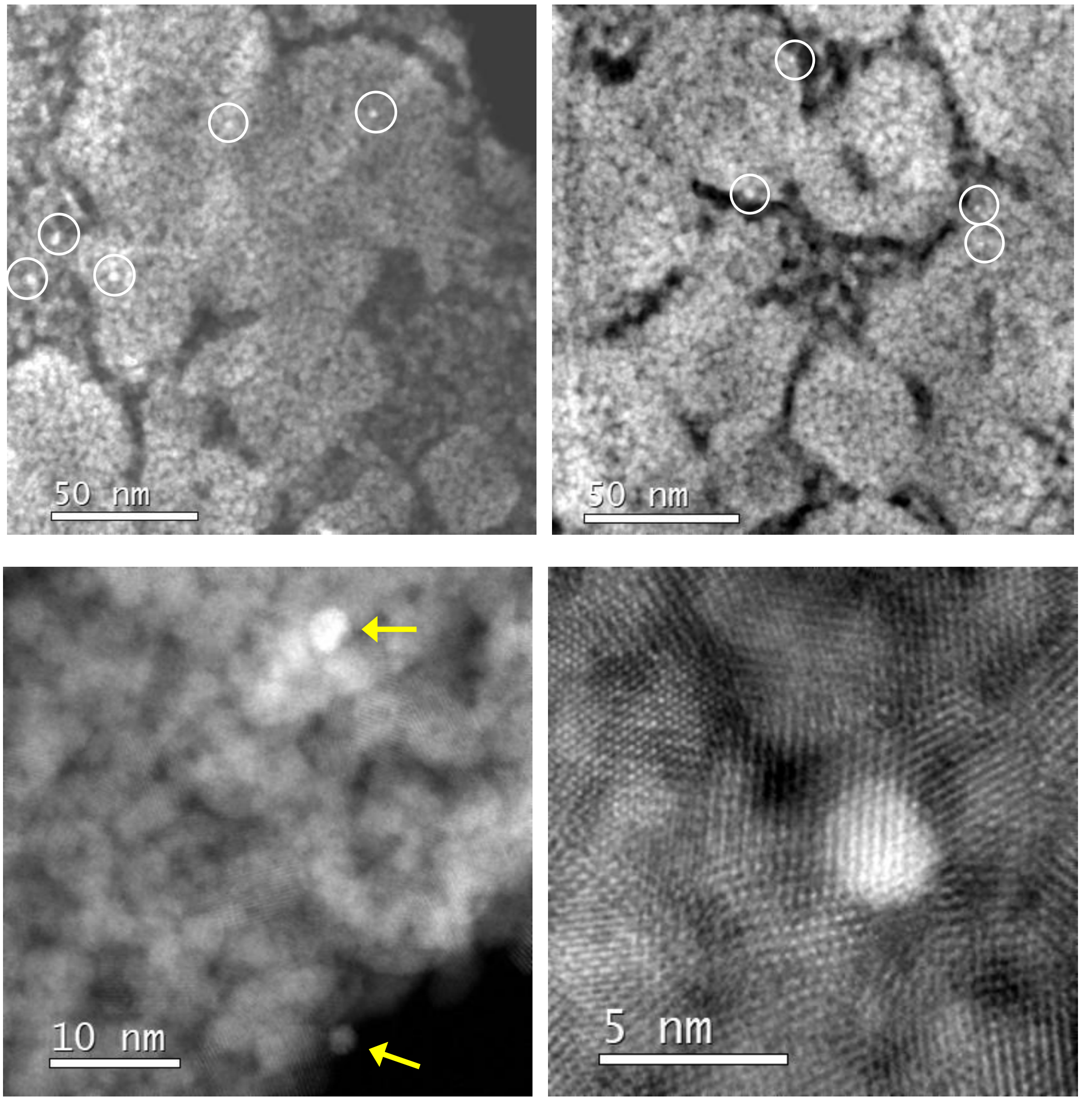

Supplementary Figure 2. Representative HAADF-STEM images of Au/CeO_2-NP with different magnifications.

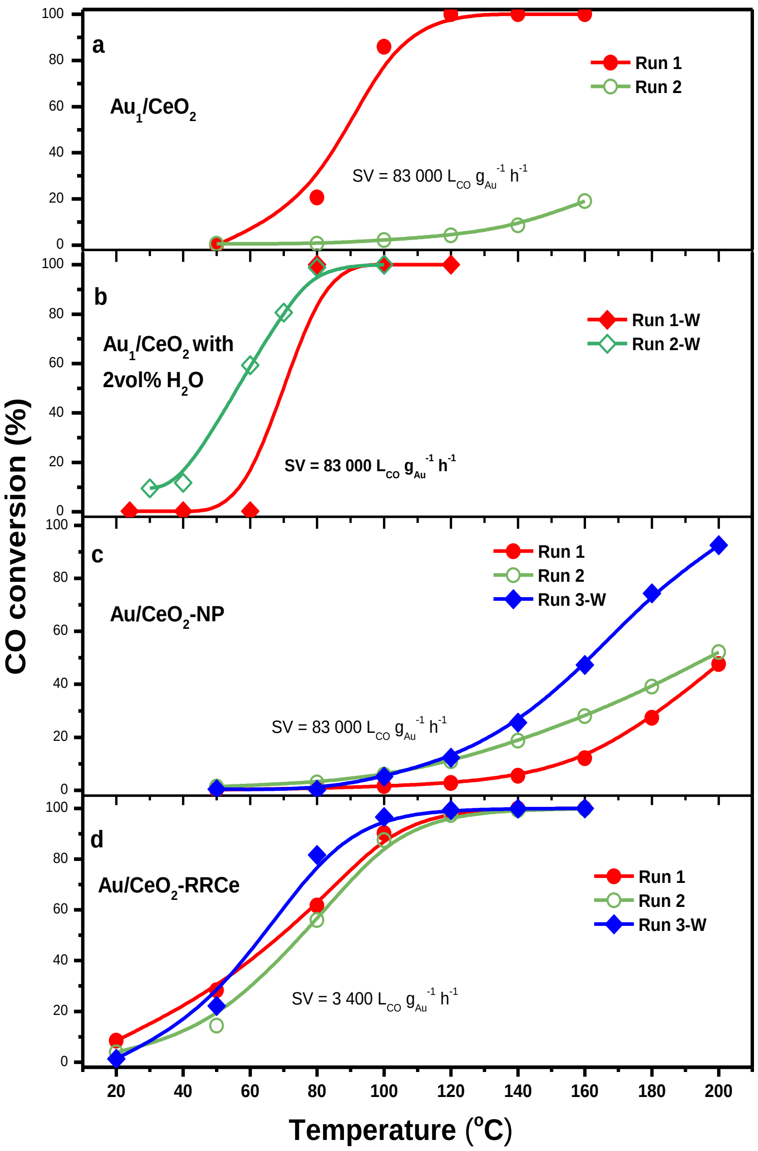

Supplementary Figure 3. CO conversion with or without the presence of H_2O (2 vol%) over different catalyst. (a) Au_1/CeO_2 (without H_2O); (b) Au_1/CeO_2 (with H_2O); (c) Au/CeO_2-NP; (d) Au/CeO_2-RR2Ce. Note that in panel (c) and (d) Run 1 and Run 2 were without H_2O and Run 3-w means the reaction run in presence of H_2O. Reaction condition: 1 vol% CO + 1 vol% O_2 (+ 2 vol% H_2O) balanced with He with a flowrate of 33.3 mL min^{-1}; 80 mg sample used (60 mg Au/CeO_2-22RCe).

Supplementary Note 1

For the Au/CeO_2-NP, a second cycle of reaction, in dry conditions (Supplementary Figure 3c) also provokes an improvement of the catalytic activity. We think that the slight activity improvement might be due to the remove of the residual stabilizer: the Au NPs were prepared by a colloidal method stabilized by PVA. Prior to testing we have performed an in situ heat-treatment of the sample at 200 ^{o}C to remove the PVA stabilizer,[1] but the presence of trace amount of residual PVA is still possible. In addition, the metal-support interaction might have been modified by the reaction gas during the first run. On the other hand, the commercial Au/CeO_2-RRCe sample, prepared by a DP method and calcined at an elevated temperature, has negligible activity changes between the two cycles.

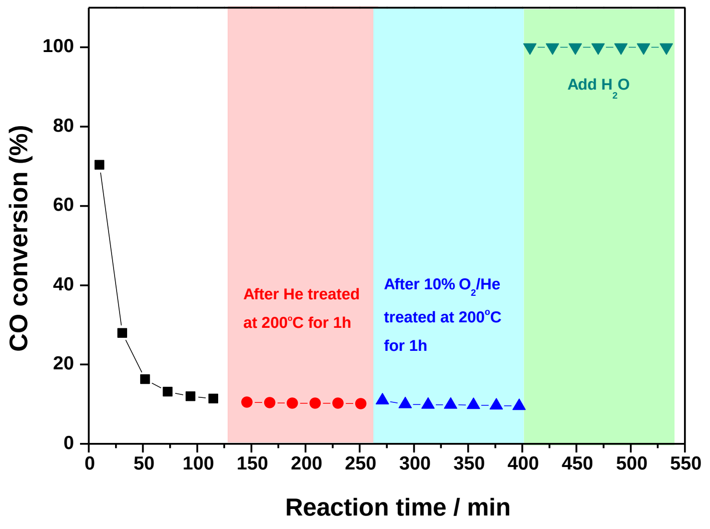

Supplementary Figure 4. CO conversion rate as a function of reaction time for CO oxidation at 200 °C on 0.03 wt% Au_1/CeO_2. Reaction conditions: 1 vol% CO + 1 vol% O_2 He balance with a flowrate of 33.3 mL min^{-1}; 20 mg catalyst was used. The flowrate of the treatment gas mixture was 33.3 ml min^{-1}; The added H_2O amount was about 2 vol%.

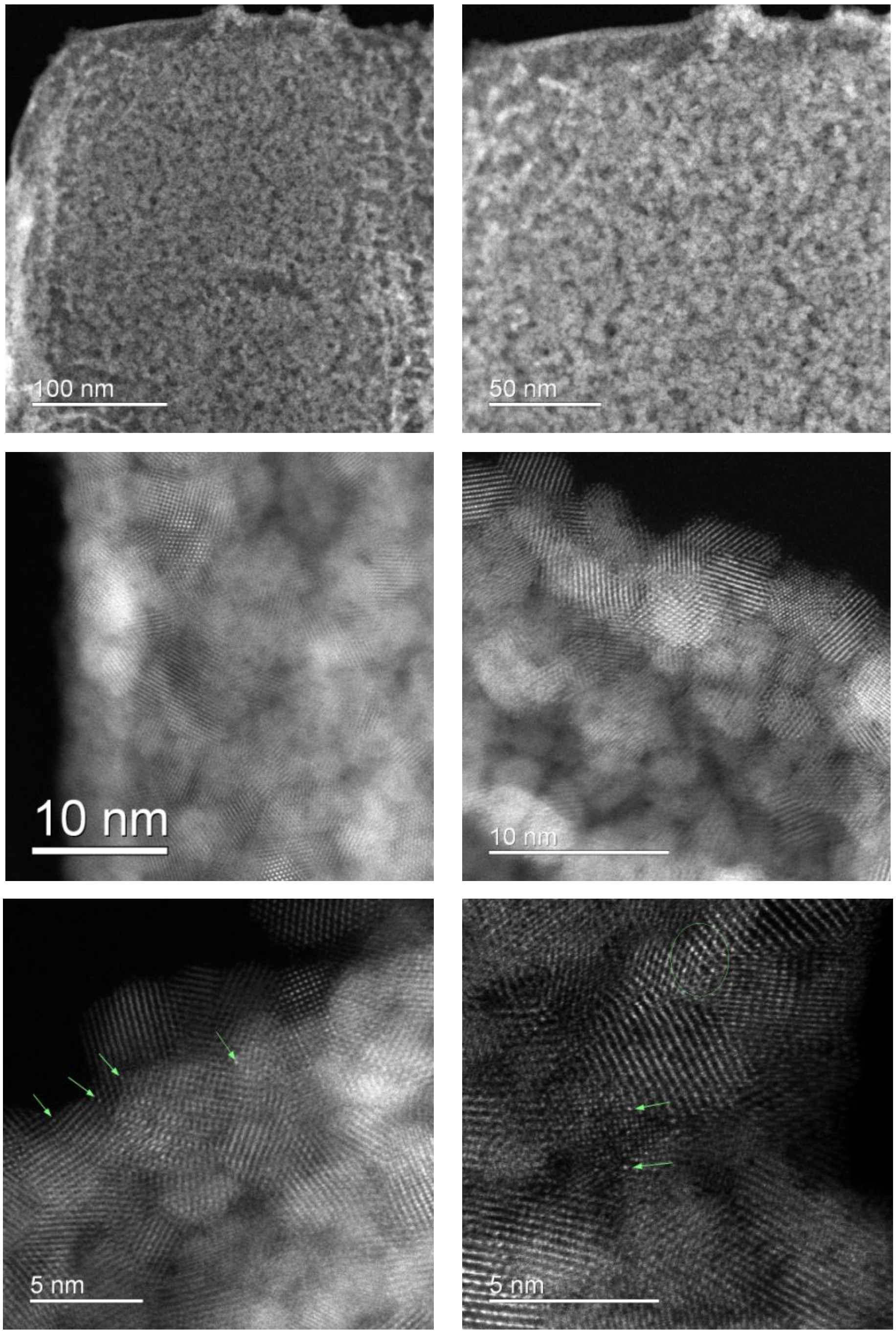

Supplementary Figure 5. Representative HAADF-STEM images of the used Au_1/CeO_2 with different magnifications.

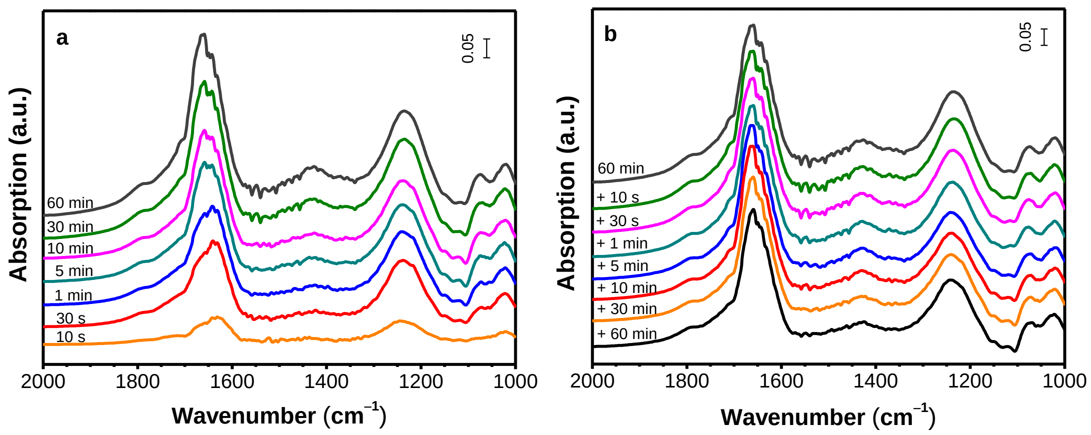

Supplementary Figure 6. In situ DRIFT spectra of Au_1/CeO_2 as a function of reaction time at 200 ^oC. (a): CO oxidation without the introduction of H_2O (reaction condition: 1 vol% CO + 2 vol% O_2 balanced He, flowrate: 50 mL min^{-1}); (b): Introducing 2 vol% H_2O into the reaction gas mixture after a.

Supplementary Note 2

As shown in Supplementary Figure 6a, for CO oxidation without the presence of H_2O, three bands appeared immediately after the reaction gas being introduced and gradually grew up with the reaction time, unambiguously demonstrating the formation of carbonates on the surfaces of CeO_2. However, the intensity of these bands kept almost unchanged after the introduction of H_2O, in contrast to the significantly increased activity and gradually recovered OH-group. The results evidenced that stable carbonates were formed during CO oxidation but did not have much effect on the activity.

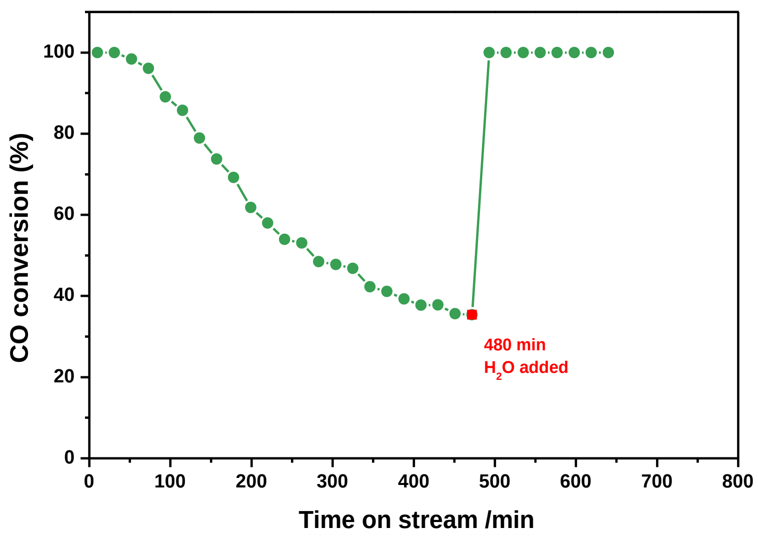

Supplementary Figure 7. CO conversion rate as a function of reaction time for CO oxidation at 160 °C on 0.03 wt% Au_1/CeO_2. Reaction condition: 1 vol% CO + 1 vol% O_2 He balance with a flowrate of 33.3 mL min^{-1}; 80 mg catalyst was used. The added H_2O amount was about 2 vol%.

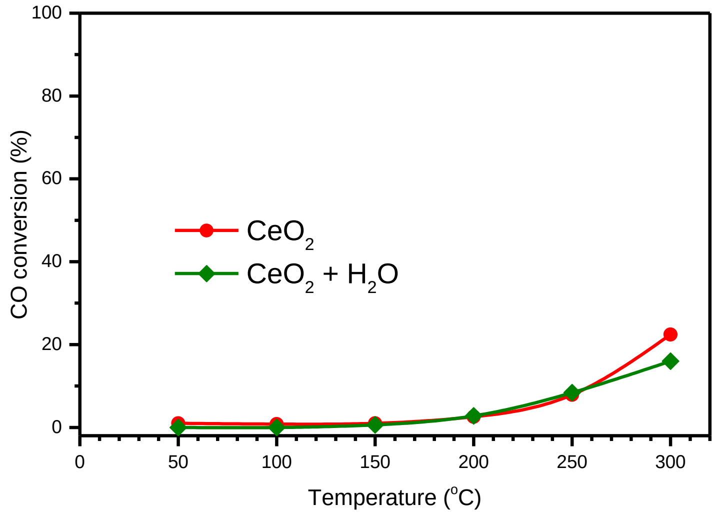

Supplementary Figure 8. CO oxidation on CeO_2 support with or without the presence of water. Reaction condition: 1 vol% CO + 1 vol% O_2 (+ 2 vol% H_2O) He balance with a flowrate of 33.3 mL min^{-1}, 80 mg CeO_2.

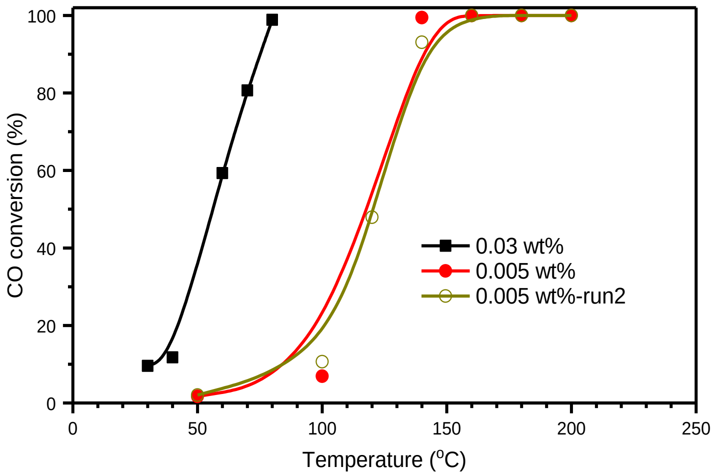

Supplementary Figure 9. CO oxidation performance with the presence of H_2O on Au_1/CeO_2 catalyst with different Au loadings. Reaction condition: 1 vol% CO + 1 vol% O_2 + 2 vol% H_2O He balance with a flowrate of 33.3 mL min^{-1}, 80 mg catalyst.

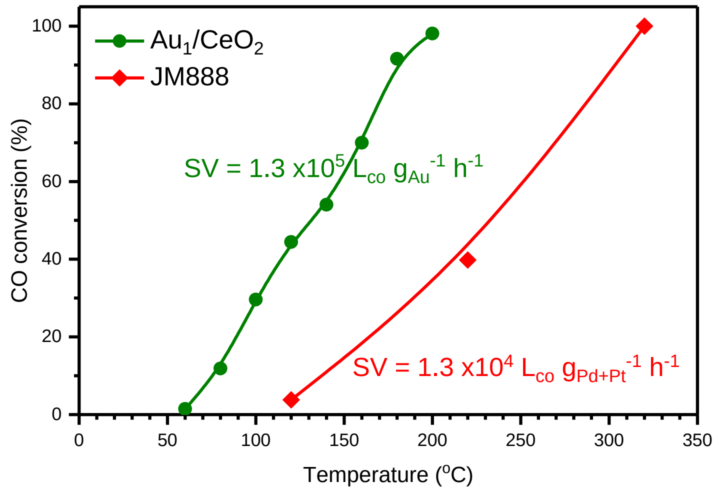

Supplementary Figure 10. CO conversion curves as a function of reaction temperature on Au_1/CeO_2 (50 mg) and TWC of JM888 (100 mg) in simulated CO emission oxidation. Reaction gas composition: 1.6 v% CO, 1 v% O_2, 0.01 v% propene, 0.0087 v% toluene, 10 v% water and balanced with He with a flowrate of 33.3 mL min^{-1}.

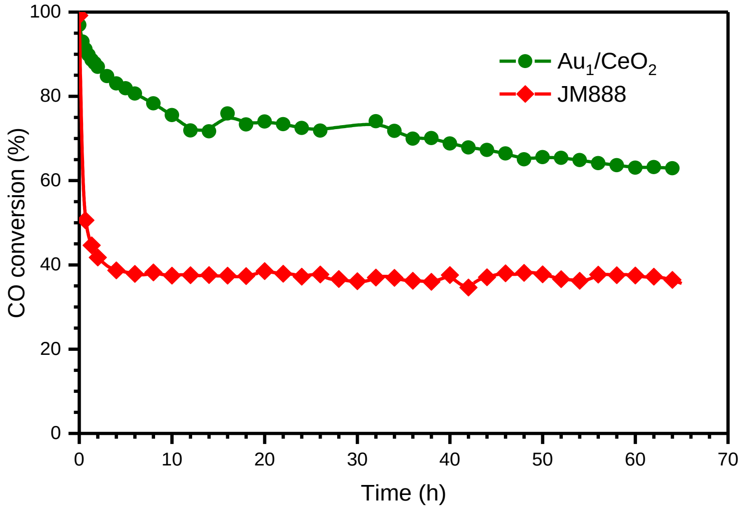

Supplementary Figure 11. CO conversion curves as a function of reaction time on Au_1/CeO_2 (40 mg) and TWC of JM888 (300 mg) at 200 ^oC. Reaction gas composition: 1.6 v% CO, 1 v% O_2, 0.01 v% propene, 0.0087 v% toluene, 10 v% water and balanced with He. For Au_1/CeO_2 with SV of 50 L g_{cat}^{-1} h^{-1} (~ 170 000 L g_{Au}^{-1} h^{-1}) and for TWC of JM888 with SV of 4 L g_{cat}^{-1} h^{-1} (2 000 L g_{Pt+Pd}^{-1} h^{-1}).

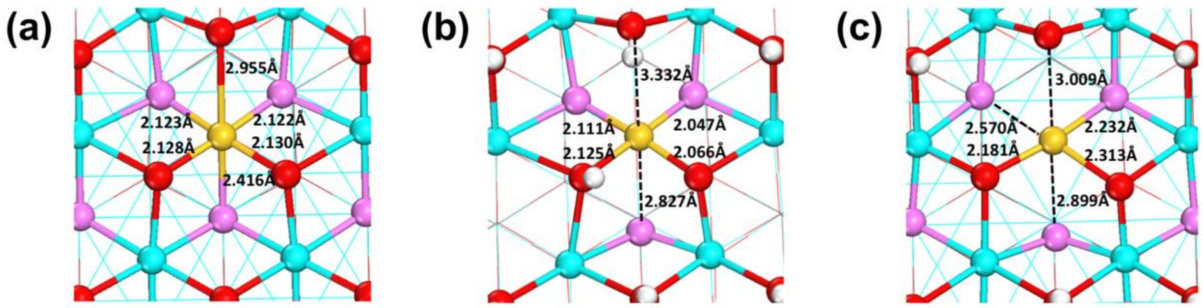

Supplementary Figure 12. Optimized structures and selected bond distances of Au_1/CeO_2 (111) surfaces. (a) Pure oxide surface, (b) hydroxylated surface with one of the three surface oxygen atoms bonded to Au atom not hydroxylated. (c) hydroxylated surface with three oxygen atoms bonded to Au atoms not hydroxylated (Ce: blue, Au: yellow, H: white, O_{surf}: red, $O_{sub\text{-}surf}$: pink).

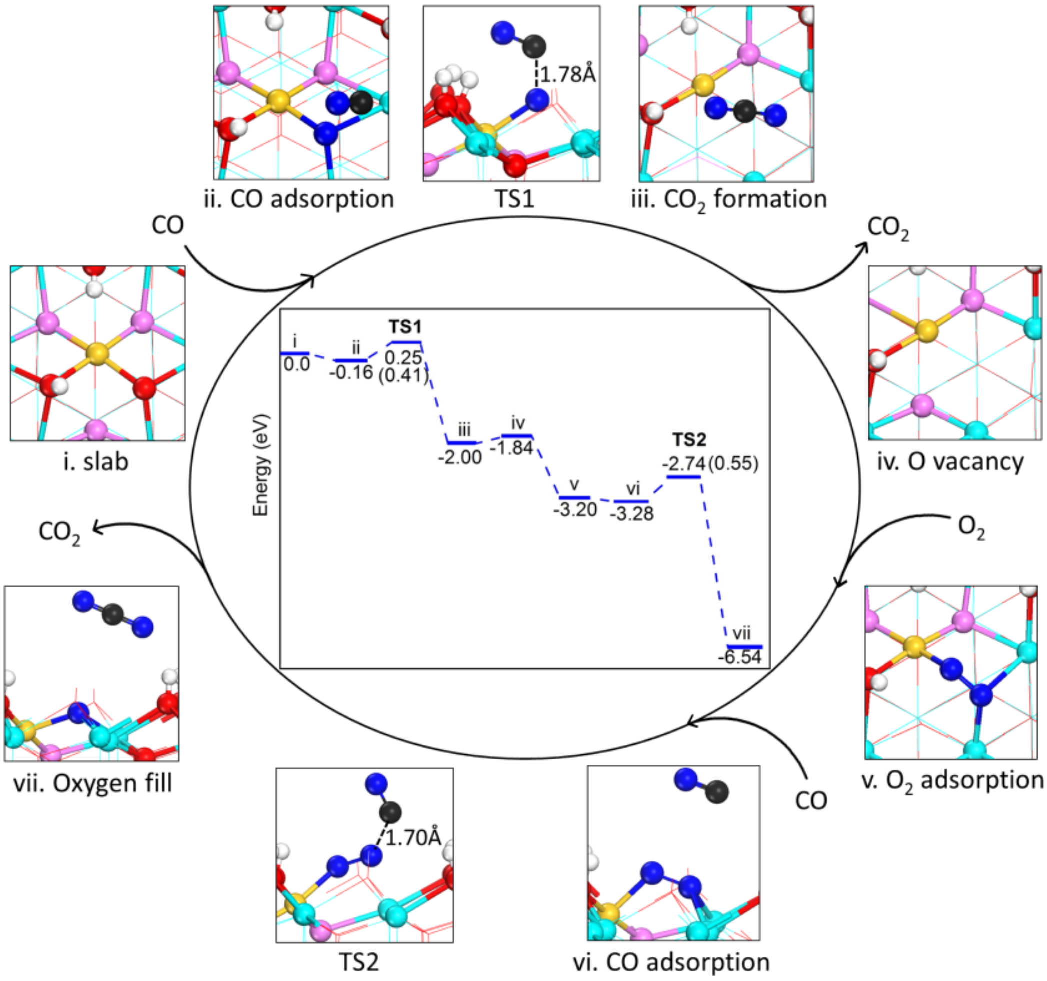

Supplementary Figure 13. Reaction pathway-A for CO oxidation on the Au_1/CeO_2 (111). The inset in the catalytic circle shows the energy profile, and numbers in the parentheses indicate the barriers of elementary steps.

Supplementary Note 3

The reaction pathway consists of three steps: (1) The oxidation of the first CO molecule with lattice oxygen leads to O vacancy formation and CO_2 desorption. The co-adsorption of CO with a lattice O neighboring the substitutional $Au^{\delta+}$ results in the direct CO oxidation with an activation barrier of 0.41 eV, and the process is exothermic by -1.84 eV. On the Au_1/CeO_2(111) surface without presence of OH groups, this process has no activation energy

and exothermic as much as -3.24 eV [2]. (2) A molecular O_2 adsorbs on the O vacancy. Once the oxygen defect is formed, it is immediately filled by an O_2 molecule from the gas phase with a binding energy of -1.36 eV. (3) The second CO molecule reacts with the adsorbed $O_2^{\delta-}$ species, releasing the second CO_2 and recovering the stoichiometry of ceria surface. The estimated barrier of this process is 0.55 eV, and highly exothermic by -3.26 eV. It is noted that the process of oxidation of the second CO with O adspecies has no activation barrier on the pure oxide surface without OH-group[3]. In addition, the oxygen vacancy formation energy of the hydroxyl covered surface is much larger than that of the pure surface (1.42 eV vs. 0.13 eV [4]).

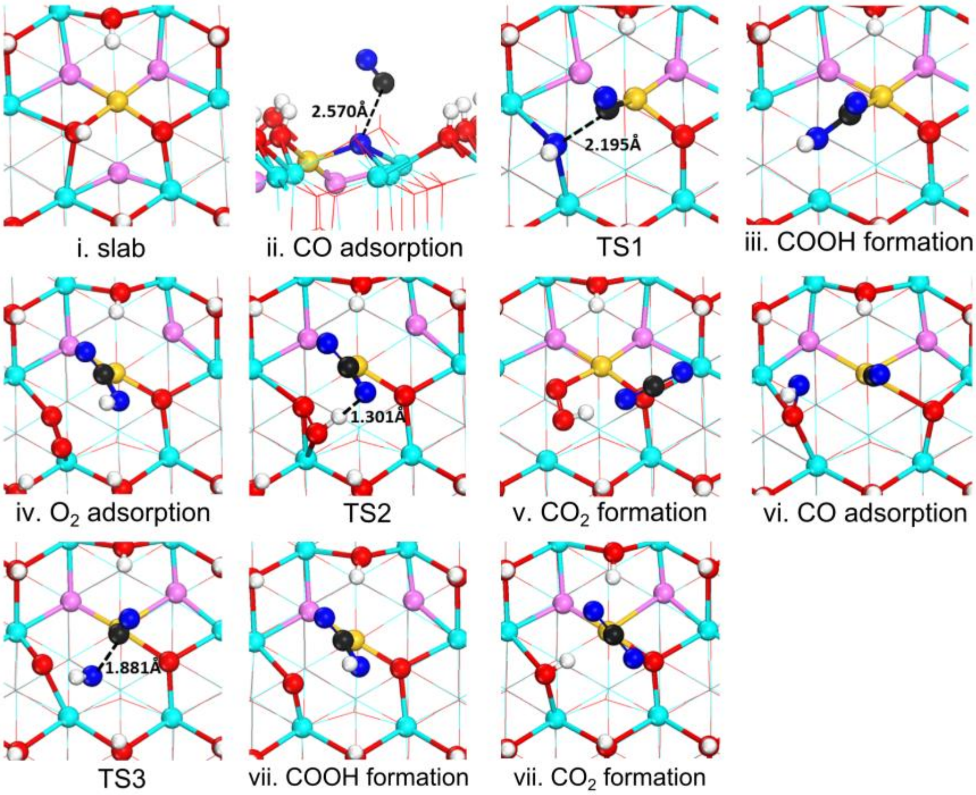

Supplementary Figure 14. Structures of key stationary intermediates involved in the Reaction pathway-B for CO oxidation on the Au_1/CeO_2 (111).

Supplementary Note 4

After adsorption of CO, the next stage in the mechanism is surface OH reacting with CO to form $COOH^*$, via a trivial transition state of just 0.02 eV, releasing 0.84 eV at this step. An oxygen vacancy is formed on the surface after the formation of the first $COOH^*$ species and it can be filled by an O_2 molecule in the gas phase with a binding energy of -0.92 eV. A very low barrier of 0.09 eV then be overcome in order to remove H from $COOH^*$ onto the adsorbed $O_2^{\delta-}$ species, in an exothermic step (-0.87 eV) that forms OOH^* and releases CO_2 into the gas phase. The newly formed OOH^* is a key intermediate for subsequent CO oxidation. The second CO molecule reacts with the OOH^* species to form $COOH^*$ and a lattice oxygen, by

overcoming a barrier of 0.07 eV and highly exothermic by -2.19 eV. Then the hydroxylated ceria surface can be recovered by releasing the second CO_2 via the H-transfer reaction ($COOH + O_L \rightarrow CO_2 + OH$). Because of the much lower barrier in the catalytic cycle, pathway-B is much more feasible than pathway-A.

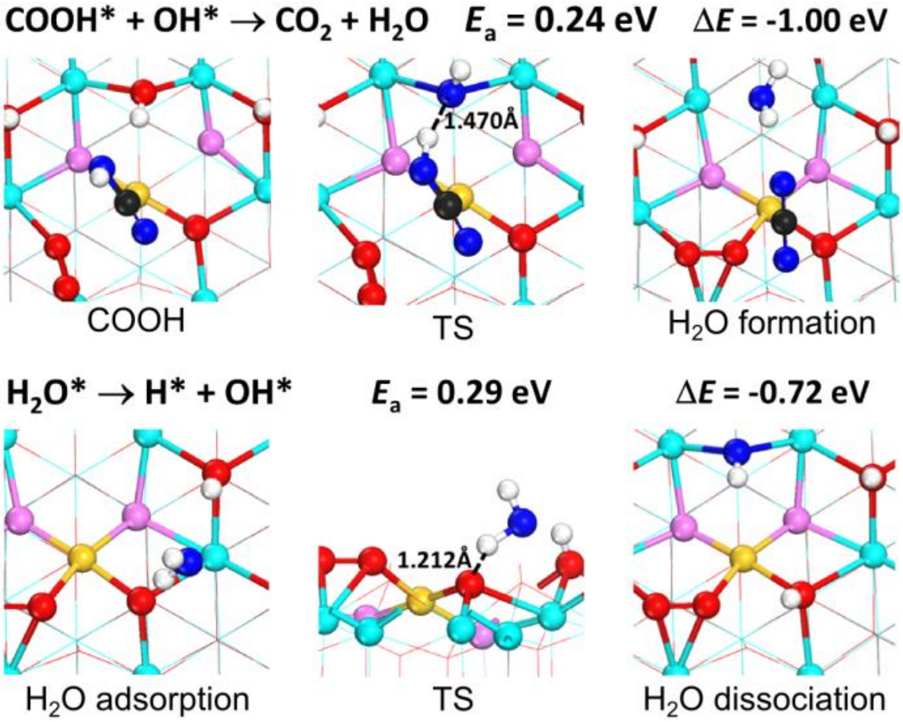

Supplementary Figure 15. Formation and dissociation of H_2O on the Au_1/CeO_2 (111). E_a is the activation barrier, and ΔE is the reaction energy.

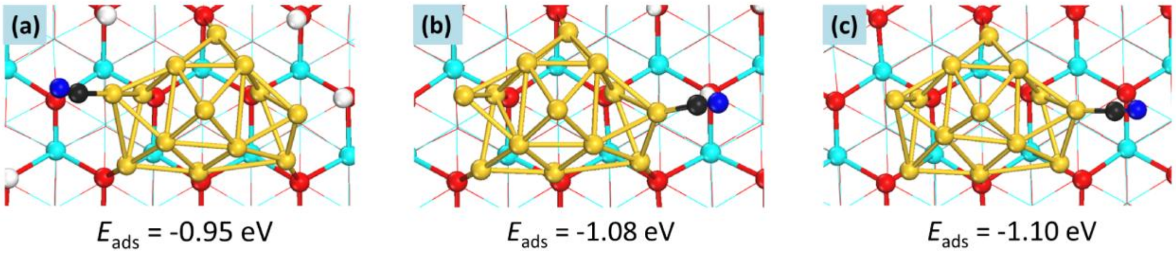

Supplementary Figure 16. Optimized structures of CO adsorbed on the Au_{13}/CeO_2 (111). E_{ads} is the adsorption energy of CO. A 4×4 CeO_2 (111) slab was used to model (a) and (b) hydroxylated surface, (c) pure oxide surface.

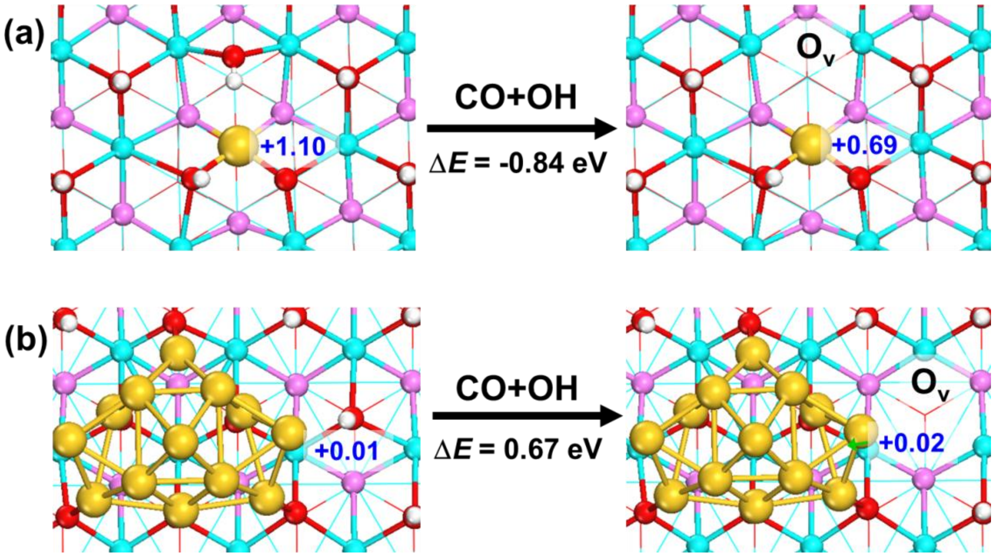

Supplementary Figure 17. Charge change of Au atoms and Au NPs as well as the reaction energy for CO + OH → COOH. (a) on Au_1/CeO_2 SAC, and (b) on Au/CeO_2-NP.

Supplementary Table 1. Catalytic performance (reaction rate and TOF) for various supported Au catalyst with or without the presence of H_2O.

	Catalysts	Au loadings (wt%)	Temperature (^oC)	Specific rate without H_2O ($mol_{CO}\ g_{Au}^{-1}\ h^{-1}$)	Specific rate with H_2O ($mol_{CO}\ g_{Au}^{-1}\ h^{-1}$)	Increment (times)*	TOF with H_2O (s^{-1})	
Entry 1	Au/CeO_2-RRCe	0.98	27	0.13	--	--	--	This work
Entry 2	Au_1/CeO_2	0.03	24	N.A.	3.6	--	0.2	This work
Entry 3	Au_1/CeO_2	0.03	100	0.82	157	190	8.6	This work
Entry 4	Au_1/CeO_2	0.03	200	9.91	1500	150	82.0	This work
Entry 5	Au /CeO_2-NP	0.03	24	N.A.	N.A.		N.D.	This work
Entry 6	Au /CeO_2-NP	0.03	100	4.0	2.5	-0.4	0.453	This work
Entry 7	Au /CeO_2-NP	0.03	200	38.1	62.6	0.6	11.4	This work
Entry 8	Au/CeO_2-RRCe	0.98	200	13.0	37.3	1.9	10.2	This work
Entry 9	Au/Fe_2O_3	4.4	200	21.3	82.2	2.8	18	This work
Entry 10	Au/TiO_2	1.5	200	161	205	0.3	45	This work

* Increment = (specific rate with H_2O - specific rate without H_2O)/ specific rate without H_2O

Supplementary Table 2. Calculated oxygen vacancy (O_v) formation energies and charges of Au for Au_1 and Au_{13} with different models.

	Corresponding models	Charge of Au |e|	Reaction heat for CO+OH → COOH (eV)	Charge of Au after CO+OH →COOH reaction |e|	Oxygen vacancy formation energy (eV)
Au_1/CeO_2	A	+1.26	--	--	0.13
Au_1/CeO_2-OH	B	+1.10	-0.84	+0.69	1.42
Au_1/CeO_2-3O	C	--	--	--	1.23
Au_{13}/CeO_2-OH	A/B	+0.01	0.67	+0.02	2.26
Au_{13}/CeO_2	C	+0.03	-	--	2.29
Au-OH		+0.372			
Au-$(OH)_2$		+0.690			
Au-$(OH)_3$		+0.988			

Supplementary Table 3. Calculated pre-exponential factors *A* (s^{-1}), activation barriers E_a (eV), and the rate constants *k* (s^{-1}) at 473 K of pathway A and B.

	Pathway A		Pathway B	
	$CO+O_L \rightarrow CO_2+O_V$	$CO+O_2^* \rightarrow CO_2+O_L$	$COOH^* + O_2^* \rightarrow CO_2 + OOH^*$	$COOH^* + OH^* \rightarrow CO_2 + H_2O$
A (s^{-1})	8.69×10^{12}	1.19×10^{10}	1.48×10^{12}	7.74×10^{13}
E_a (eV)	0.41	0.55	0.09	0.24
k (s^{-1})	3.77×10^{8}	1.82×10^{4}	1.73×10^{11}	2.43×10^{11}

Supplementary References

1. Comotti M, Li W-C, Spliethoff B, Schüth F. Support Effect in High Activity Gold Catalysts for CO Oxidation. *J Am Chem Soc* **128**, 917-924 (2006).

2. Camellone MF, Fabris S. Reaction mechanisms for the CO oxidation on Au/CeO_2 catalysts: Activity of substitutional Au^{3+}/Au^{+} cations and deactivation of supported Au^{+} adatoms. *J Am Chem Soc* **131**, 10473-10483 (2009).

3. Qiao B*, et al.* Highly Efficient Catalysis of Preferential Oxidation of CO in H2-Rich Stream by Gold Single-Atom Catalysts. *ACS Catalysis* **5**, 6249-6254 (2015).

4. Zhao Y, Teng B-T, Wen X-D, Zhao Y, Zhao L-H, Luo M-F. A theoretical evaluation and comparison of MxCe1−xO2−δ (M=Au, Pd, Pt, and Rh) catalysts. *Catal Commun* **27**, 63-68 (2012).
