# Supplementary material for: Remarkable active-site dependent H2O promoting effect in CO oxidation
Source: Nat Commun. 2019 Aug 23;10:3824. doi: 10.1038/s41467-019-11871-w (PMC6707188; doi:10.1038/s41467-019-11871-w)
Supplement: Supplementary file 2 — Peer Review [file 41467_2019_11871_MOESM2_ESM.pdf]

Reviewers' comments:

Reviewer #1 (Remarks to the Author):

The subject treated in this paper is very interesting in the catalysis field: the role of water in oxidation reactions. The authors analyse the catalytic activity of SACs (Au_1/CeO_2) and Au/CeO_2 (gold nanoparticles) in the CO oxidation in the presence of water. Results evidence that water has a promoting effect in SAC more important than in the case of interfacial sites of gold nanoparticles catalysts. Moreover, computational studies were carried out and permitted to attribute the special behaviour of SAC to the electronic structure and local geometry of gold atoms in this system. Finally, the authors propose that the reaction proceeds with the participation of OH groups generated in the presence of water.

Despite the great interest of this work, I have some comments and suggestions.

Figure 1 shows the CO conversion as a function of the reaction time. In this, a rapid deactivation of the catalyst when the reaction is carried out at 200ºC in absence of water is observed. The authors think that carbonate species are not responsible for this deactivation because the reaction temperature is high. Are you sure that the catalysts surface is free of carbonates in these conditions?? Some types of cerium carbonates show a very high thermal stability. The absence of carbonates on the surface can be easily demonstrated by inspection of DRIFT spectra. Moreover, in figure S3-A, the reaction is carried out in dry conditions, but no deactivation is observed even at very high conversions at 120-160ºC. Why if the temperature is lower than 200ºC?

Figure S3-B shows the CO conversion as a function of the time for two cycles of reaction in similar conditions (with water) and it is observed the improvement of the catalytic activity in the second one. In my opinion, a modification of the catalytic behaviour of a solid in consecutive reaction cycles, means that the surface (species, electronic density, …) has been modified during the first one. What happens in the case of Au_1/CeO_2? For the Au/CeO_2-NP, a second cycle of reaction, in dry conditions (figure S3-C) also provokes an improvement of the catalytic activity. I think that a detailed discussion of these aspects is needed in the paper.

In addition, the paper is focused to demonstrate that water influences the catalytic activity of SAC in a different way than the interfacial sites in gold nanoparticles. However, the catalytic reactions have been carried out in a mixture $CO+O_2$, what is the role of oxygen? The authors attribute the Au_1/CeO_2 deactivation in dry conditions to the consumption of OH species. This idea suggests that oxygen has not effect on the CO oxidation reaction, but it is not the case. This point has to be discussed in order to better understand the reaction. The oxygen activation in the CO oxidation reaction has been a very discussed point in the literature, and therefore, I suggest the discussion of this aspect.

An operando DRIFTS analysis has also been carried out in order to detect the modification during the reaction. Figure 4 shows difference spectra recorded during the reaction without and with water. In this, bands of CO in gas phase are overlapping other possible species such as the typical band due to an electronic transition in Ce^{3+}, around 2130 cm^{-1}. The authors try to demonstrate that Ce^{4+} is reduced during the “dry” reaction while after the water introduction, the Ce^{3+} is oxidized to Ce^{4+}. In this analysis, a band at 2089 cm^{-1} (hardly observed due to the presence of CO in gas phase) has been attributed to CO adsorbed on Ce^{3+}. I don’t agree with this attribution, because a carbonyl bands appearing at wavenumbers lowers than 2143 cm^{-1}, reflects the presence of sites with a very high backdonation ability (high electronic density), and this is not the case for Ce^{3+} species. The attribution of this band has to be considered. Moreover, the attribution of OH species should be revised.

Reviewer #2 (Remarks to the Author):

The authors report on the results of an experimental and theoretical study on the promoting effect of water on single-atom Pt-CeO2 catalysts. They show that the Pt-CeO2 single atom catalyst deactivates quickly at elevated temperatures in the absence of water. In contrast, the activity is rapidly restored by adding water to the feed. The experimental results are rationalized on the basis of DFT calculations. It is suggested that, in the absence of water, CO oxidation proceeds via a MvK mechanism (involving lattice oxygen) while, in the presence of water, the CO oxidation mechanisms directly involves OH. The authors address in intensely discussed topic and the experimental and theoretical results are convincing. The proposed mechanism is new and consistent and the proposed difference between the Au NPs and the Au SACs are reasonable. In this respect, the present work represents a major step forward in this field. Also, the manuscript is concise and well written. I believe that the present work should be published after minor revisions:

1. The principal issue in the present study (similar to many other pieces of work that combine catalytic tests on real catalysts with DFT modelling) is the choice of the model system. How much is actually known from experiment on the atomistic surface structure of the materials? There is an intrinsic risk that the surface models are tested one by one until a reasonable correspondence is found with the experimental trends (which does neither imply that the model is correct nor that it is unique). The authors will not be able to solve this issue, but they should at least try to motivate the choice of the surface model properly. What is known on the surface structure, composition, and stability of the CeO2-SAC which models are in principle possible and how is the present one derived?
2. Figure 2: From which data was the CO oxidations rate calculated? If it is the data shown in the SI, the results are not too meaningful. The conversion is at 100% for all samples and, therefore, the rates are limited by the experimental conditions and not by the catalyst. This will not allow for a proper comparison of the activities. It would be preferable to record data under conditions where no only partial conversion is achieved. In general, it would be nice to have the catalytic data from Figure S3 in the main text and not only in the SI.
3. In Figure S3, the light-off curves are given for all three catalysts, but the effect of water is only shown for the Pt-CeO2-SAC. For completeness the effect of water should also be shown for the other two catalysts.
4. L42/43: "…catalysts because the interfacial perimeter sites usually serve as the dominant active sites." This is certainly not true in general. In fact, there are many cases, in which the boundary sites is not the most active .
5. L. 31/32: "…due to the quantum size and ensemble effects of metal nanoparticles…". Talking about heterogeneous catalysis in general, there are other effects that may play a role, for example the electronic and support effects discussed in this work.
6. Figure 3: the arrow for CO2 has the wrong orientation (against the catalytic cycle).
7. Line 267: "which makes the reaction exothermic", change to "which makes the reaction step exothermic" (the overall thermodynamics is not changed).

Reviewer #3 (Remarks to the Author):

This work analyses the catalytic performances of a promising catalyst Au1/CeO2, that was already proposed by a subset of authors (see Ref. 19, ACS Catalysis 2015), and that displays good performances towards CO oxidation as compared to commercial systems. In particular here the authors proposes that the fast deactivation of these Au/CeO2 catalysis originates from the consumption of OH species rather than from the sintering of the Au1 adatoms. In my opinion this is an interesting well-written article, that however presents a series of issues that I would ask the authors to address before I can give a recommendation on this work. On a more general level, I find that the main message, although interesting and deserving to be published on a good journal, could be better suited to a more specialised journal (i.e. catalysis).

1. The authors do not address at all the issue of sintering of Au species. I think that they should provide a more solid evidence that sintering can be excluded.
2. The nomenclature and charge of the Au1/CeO2 system is puzzling. As far as I understand the Au1 atom is substitutional for a surface Ce atom. The system is therefore more similar to a solid solution than to a supported catalyst. This could explain why sintering is not observed, although - again - the authors should clarify it (see point 1). What is puzzling is the charge of this Au1 atom, reported to be "positively charged, non-zero valentine’s Au^{\delta +} atom. In the literature, this notation implicitly suggest a small positive charge. Instead, for a solid solution, the ionic nature of the Au1 species should be prominent, with oxidation states close to 3+ or 4+, as previously reported on the basis of photoemission spectroscopy. This issue should in my opinion made clear, and some experimental data assessing the charge should be presented.
3. The computational study model the Au1/CeO2 system on the basis of a substitutional Au atom on a flat (111) surface. The authors should provide more solid evidence that this highly ideal model does capture the physical features of the new catalyst. For example, the Au atom in this model will be highly ionic (see point 2).

Response to reviewers' comments and questions

Reviewer #1 (Remarks to the Author):

The subject treated in this paper is very interesting in the catalysis field: the role of water in oxidation reactions. The authors analyse the catalytic activity of SACs (Au1/CeO2) and Au/CeO2 (gold nanoparticles) in the CO oxidation in the presence of water. Results evidence that water has a promoting effect in SAC more important than in the case of interfacial sites of gold nanoparticles catalysts. Moreover, computational studies were carried out and permitted to attribute the special behaviour of SAC to the electronic structure and local geometry of gold atoms in this system. Finally, the authors propose that the reaction proceeds with the participation of OH groups generated in the presence of water.

Despite the great interest of this work, I have some comments and suggestions.

Question (1): *Figure 1 shows the CO conversion as a function of the reaction time. In this, a rapid deactivation of the catalyst when the reaction is carried out at 200ºC in absence of water is observed. The authors think that carbonate species are not responsible for this deactivation because the reaction temperature is high. Are you sure that the catalysts surface is free of carbonates in these conditions?? Some types of cerium carbonates show a very high thermal stability. The absence of carbonates on the surface can be easily demonstrated by inspection of DRIFT spectra. Moreover, in figure S3-A, the reaction is carried out in dry conditions, but no deactivation is observed even at very high conversions at 120-160ºC. Why if the temperature is lower than 200ºC?*

Response: We thank your nice comments on the value of our research work and we also appreciate your insightful questions that help us to clarify our discussions in the revised manuscript.

We agree with you that during the CO oxidation reaction the formation of carbonate species on CeO_2 surfaces can occur, especially at low reaction temperatures. In order to unambiguously identify the sources of the observed deactivation we have now checked the CO adsorption band region of 1200-1800 cm^{-1} (carbonates region) by conducting operando FT-IR with and without the presence of H_2O in the reactant gas mixture. As shown below in Figure R1A, for CO oxidation without the presence of H_2O, three bands appeared immediately after the reaction gas being introduced and the intensity of these bands gradually increased with the reaction time, unambiguously demonstrating the formation of carbonates on CeO_2 surfaces. However, the intensity of these bands kept almost unchanged when H_2O was introduced, in contrast to the significant increase of intensity of the OH-group with time. These results proved that stable carbonates were indeed formed during CO oxidation but they did not change appreciably when H_2O was introduced or eliminated. Therefore, we propose that the presence of the carbonates on the CeO_2 surfaces did not cause the observed

deactivation.

In order to further confirm this hypothesis we have performed in situ heat-treatment of the deactivated catalyst by He and O_2 at 200 ^oC for 1 h. Such treatment has been proved effective (at least partially) to remove the carbonates and recover the catalyst activity for CO oxidation (*Nat Chem* 2011, *3* (8), 634, *ACS Catal.* 2014, *4* (7), 2113). Figure R2 shows clearly that both the He and O_2 treatments have no effect on the recovery of the CO conversion rate. The CO oxidation activity, however, increased dramatically after the introduction of a small amount of water. These new experimental results further corroborate our conclusion that stable carbonate species indeed formed during the CO oxidation reaction but the presence of these carbonate species do not seem to be responsible for the experimentally observed rapid deactivation and reactivation of our Au_1/CeO_2 SAC catalyst.

As to the catalyst behavior in Figure S3a, we believe that the deactivation occurred during the CO oxidation process. The rate of deactivation is not obvious because of i) the catalyst may deactivate slower at temperatures lower than that at 200 ^oC (The consumption of the OH groups is higher at higher temperatures) and ii) the fact that the CO conversion rate increases with reaction temperature offsets the deactivation with reaction time. Therefore, it is difficult to evaluate the deactivation rate by simply examining the "conversion vs. reaction temperature" plot. To confirm this hypothesis, we have conducted a stability test at 160 ^oC. As shown in Figure R3, the catalyst certainly deactivated but indeed experienced a slower deactivation process compared with that conducted at 200 ^oC. In addition, the introduction of water immediately recovers the activity, similar to that at 200 ^oC. We have added the new data and discussions in pages *4-6* of the revised manuscript and pages *5-9* of the updated supporting information (Figure S4, S6 and S7).

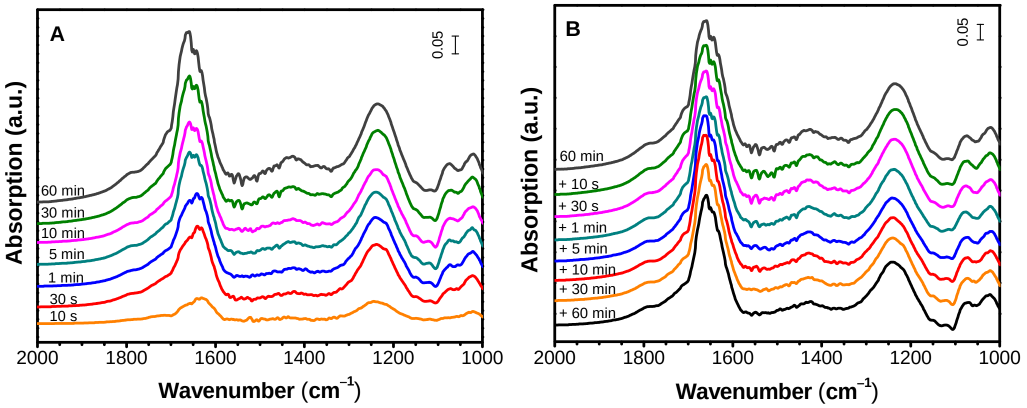

Figure R1 In situ DRIFT spectra of Au_1/CeO_2 as a function of reaction time at 200 ^oC. A: CO oxidation without the presence of H_2O (reaction condition: 1 vol% CO + 2 vol% O_2 balanced He, flowrate: 50 mL/min); B: Introducing 2 vol% H_2O into the reaction gas mixture after A.

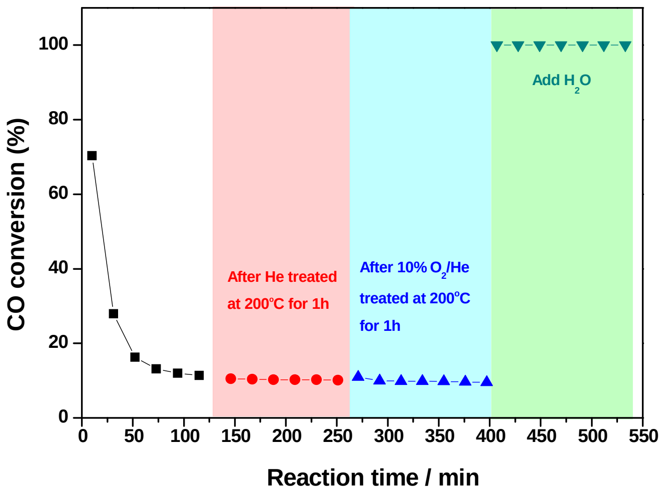

Figure R2 CO conversion rate as a function of reaction time for CO oxidation at 200 °C on 0.03 wt% Au_1/CeO_2. Reaction conditions: 1 vol% CO + 1 vol% O_2 He balance with a flowrate of 33.3 mL/min; 20 mg catalyst was used. The flowrate of treated atmosphere is 33.3ml/min; The added H_2O amount is about 2 vol%.

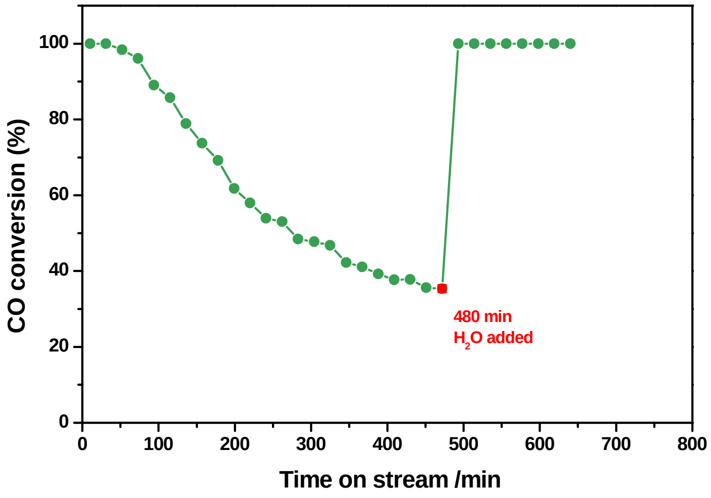

Figure R3 CO conversion rate as a function of reaction time for CO oxidation at 160 °C on 0.03 wt% Au_1/CeO_2. Reaction condition: 1 vol% CO + 1 vol% O_2 He balance with a flowrate of 33.3 mL/min; 80 mg catalyst was used. The added H_2O amount is about 2 vol%.

Question (2): *Figure S3-B shows the CO conversion as a function of the time for two cycles of reaction in similar conditions (with water) and it is observed the improvement of the catalytic activity in the second one. In my opinion, a modification of the catalytic behaviour of a solid in consecutive reaction cycles, means that the surface (species, electronic density, …) has been modified during the first one. What happens in the case of Au1/CeO2? For the Au/CeO2-NP, a second cycle of reaction, in*

dry conditions (figure S3-C) also provokes an improvement of the catalytic activity. I think that a detailed discussion of these aspects is needed in the paper.

Response: The reviewer raised an excellent suggestion. We agree with the reviewer that the catalyst might have been slightly modified by the reaction gas (*Chin J Catal* **2015,** *36* (9), 1505-1511) and/or by the presence of H_2O vapor during the first run. Especially the latter can have dramatic promoting effect on the catalytic performance of Pt_1/CeO_2 SAC as evidenced recently in an elegant work (*Science* **2017,** *358* (6369), 1419-1423). In addition, considering that our Au_1/CeO_2 SAC was dried at 60 ^oC without further calcination at elevated temperatures, a subtle change in catalyst (surface) structure as well as metal-support interaction between the Au atoms and the CeO_2 surfaces might have also occurred during the first run. However, because the changes might be subtle and the loading of Au is low as well as the difficulty in determining variations of metal-support interactions, a detailed characterization and description of the reaction-induced changes of such catalysts seems extremely difficult, if not impossible, with the current technique.

As to the Au/CeO_2-NP catalyst, we think the slight activity improvement might be due to the removal of the residual stabilizer: the Au NPs were prepared by a colloidal method stabilized by PVA. Prior to catalytic testing, we have performed an *in situ* heat treatment of the sample at 200 ^oC to remove the PVA stabilizer (*J. Am. Chem. Soc.* **2006,** *128* (3), 917) but the presence of trace amount of residual is still possible. In addition, the metal-support interaction might have been modified by the reaction gas during the first run. On the other hand, the commercial Au/CeO_2-RRCe catalyst prepared by a DP method and calcined at an elevated temperature showed negligible activity change between the two cycles. We have added the relevant discussions on page 6 of the revised manuscript and page 5 of the updated supporting information.

Question (3): *In addition, the paper is focused to demonstrate that water influences the catalytic activity of SAC in a different way than the interfacial sites in gold nanoparticles. However, the catalytic reactions have been carried out in a mixture CO+O2, what is the role of oxygen? The authors attribute the Au1/CeO2 deactivation in dry conditions to the consumption of OH species. This idea suggests that oxygen has not effect on the CO oxidation reaction, but it is not the case. This point has to be discussed in order to better understand the reaction. The oxygen activation in the CO oxidation reaction has been a very discussed point in the literature, and therefore, I suggest the discussion of this aspect.*

Response: We appreciate the reviewer's question and suggestions. We have indeed demonstrated in this work that water have distinctly different promoting effect on the CO oxidation on Au_1/CeO_2 SAC

from that on Au/CeO_2-NP catalysts through a series of experiments. However, this result just suggests that on the Au_1/CeO_2 SAC the OH-group, by working synergistically with O_2, can significantly promote the CO oxidation activity. Our work, however, does NOT mean that O_2 has no effect on the CO oxidation. To make this point clearer, we have tested the catalytic performance in the presence of H_2O without O_2, i.e., actually a water-gas shift (WGS) reaction. The reaction rate (as well as TOF) is a few times lower than that for dry oxidation, suggesting that O_2 indeed plays a critical role even in the presence of H_2O. From the DFT results, O_2 plays different role for different reaction pathways: under the dry reaction condition, the CO oxidation on the Au_1/CeO_2 SAC follows a MvK mechanism so the role of O_2 is mainly to replenish the O. Under the wet reaction condition, the CO oxidation follows a CO + OH → COOH route. In this case, the role of O_2 is mainly to help decomposing COOH into CO_2 and H. In addition, we believe that O_2 can also help the activation of H_2O. We have added this discussion on (page 11) in the revised manuscript.

Question (4): *An operando DRIFTS analysis has also been carried out in order to detect the modification during the reaction. Figure 4 shows difference spectra recorded during the reaction without and with water. In this, bands of CO in gas phase are overlapping other possible species such as the typical band due to an electronic transition in Ce3+, around 2130 cm-1. The authors try to demonstrate that Ce4+ is reduced during the "dry" reaction while after the water introduction, the Ce3+ is oxidized to Ce4+. In this analysis, a band at 2089 cm-1 (hardly observed due to the presence of CO in gas phase) has been attributed to CO adsorbed on Ce3+. I don't agree with this attribution, because a carbonyl bands appearing at wavenumbers lowers than 2143 cm-1, reflects the presence of sites with a very high backdonation ability (high electronic density), and this is not the case for Ce3+ species. The attribution of this band has to be considered. Moreover, the attribution of OH species should be revised.*

Response: We appreciate the reviewer's comments and question. First of all, although this peak is difficult to detect, we are pretty sure that the observed peak DOES exist because in several repeated measurements the phenomenon of appearance and disappearance of this small peak with removal and introduction of water were all observed. Secondly, we can rule out the possibility of assigning the peak to CO adsorption on Au atoms because the Au loading level is too low to provide detectable CO adsorption peaks. We had tried CO adsorption on 0.05 wt% Au_1/CeO_2 at room temperature but did not detect reasonable signal. Furthermore, CO adsorption on Au^0 usually yields a band at 2100-2110 cm^{-1} but on positively charged $Au^{\delta+}$ the band should be at even higher frequencies (2120-2150 cm^{-1}), which is similar to or higher than that on Ce^{3+}. Based on these reasons, we believe that ascribing this band to CO adsorption on Ce^{3+} instead of $Au^{\delta+}$ is a more reasonable assignment. To further support this assignment we performed a DFT calculation of CO adsorption on our hydroxylated CeO_2 surface and

found that the adsorption band exists at a frequency of 2072 cm^{-1}, very close to the experimentally observed band at 2089 cm^{-1} when considering that the used GGA functional usually underestimate frequencies. The DFT calculation suggests that such a frequency of CO adsorption on Ce is theoretically possible. All the above analyses indicate that our assignment is not unreasonable. However, in the revised manuscript we have changed the text into "tentatively ascribed this band at 2089 cm^{-1} to CO adsorption on Ce^{3+} on hydroxylated CeO_2 surfaces.".

As to the assignment of the OH group (3667 cm^{-1}), we do not seem to see any issues. There is a systemic study on the IR spectrum of CeO_2 (*J. Chem. Soc., Faraday Trans.* **1989,** *85*(6), 1451-1461), and the isolated OH group (3662 cm^{-1}) has been clearly identified. Other studies have the same assignment (*ACS Catal.* **2014,** *4* (6), 2088-2096,).

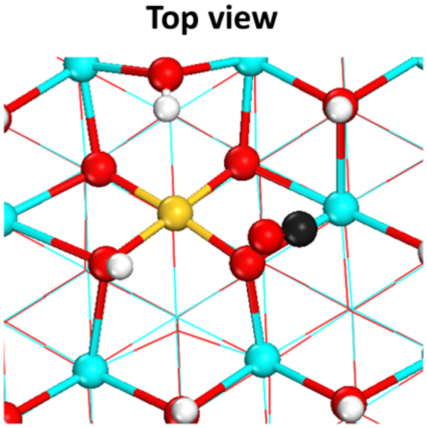

Figure R4 Top and side view of CO adsorbed on Au_1/CeO_2-OH surface.

Reviewer #2 (Remarks to the Author):

The authors report on the results of an experimental and theoretical study on the promoting effect of water on single-atom Pt-CeO2 catalysts. They show that the Pt-CeO2 single atom catalyst deactivates quickly at elevated temperatures in the absence of water. In contrast, the activity is rapidly restored by adding water to the feed. The experimental results are rationalized on the basis of DFT calculations. It is suggested that, in the absence of water, CO oxidation proceeds via a MvK mechanism (involving lattice oxygen) while, in the presence of water, the CO oxidation mechanisms directly involves OH.

The authors address in intensely discussed topic and the experimental and theoretical results are convincing. The proposed mechanism is new and consistent and the proposed difference between the Au NPs and the Au SACs are reasonable. In this respect, the present work represents a major step forward in this field. Also, the manuscript is concise and well written. I believe that the present work should be published after minor revisions:

1. The principal issue in the present study (similar to many other pieces of work that combine catalytic tests on real catalysts with DFT modelling) is the choice of the model system. How much is actually known from experiment on the atomistic surface structure of the materials? There is an intrinsic risk that the surface models are tested one by one until a reasonable correspondence is found with the experimental trends (which does neither imply that the model is correct nor that it is unique). The authors will not be able to solve this issue, but they should at least try to motivate the choice of the surface model properly. What is known on the surface structure, composition, and stability of the CeO2-SAC which models are in principle possible and how is the present one derived?

Response: We thank the reviewer for the nice comments on our work and the critical questions that help us to improve our manuscript. First of all, the possibility (or risk) of testing surface models one by one until a reasonable correspondence could be ruled out because it is not the case and would be extremely time-consuming even if one likes to. The model selection is generally on the basis of the experimental results as well as the stability of the surface. For example, if we have a given structure or a specific exposed surface, it is easy to make the model. However, in our case, unfortunately the CeO_2 was synthesized by a co-precipitation method and existed in a small nanoparticle form thus no preferentially exposed surface can be identified. In this case, we have to think about the energetically most stable structure and/or relevant reference data. In our case the reason that we had chosen CeO_2 (111) is because i) there are a good number of examples on calculating the Au single atoms on CeO_2 before (*J Am Chem Soc* **2009**, 131, 10473-10483, *J. Am. Chem. Soc.* **2017**, 139, 6190–6199) and ii) the CeO_2(111) surface is the most stable (111) surface of ceria. Therefore during the catalyst formation, the thermodynamics and kinetics make the most stable (111) surface dominant. We have added this information into the revision in the page 9 of the revised manuscript.

2. Figure 2: From which data was the CO oxidations rate calculated? If it is the data shown in the SI, the results are not too meaningful. The conversion is at 100% for all samples and, therefore, the rates are limited by the experimental conditions and not by the catalyst. This will not allow for a proper comparison of the activities. It would be preferable to record data under conditions where no only partial conversion is achieved. In general, it would be nice to have the catalytic data from Figure S3 in the main text and not only in the SI.

Response: We appreciate the reviewer's question and comments. We apologize for not making this point clearer in the original manuscript. All specific reaction rates were measured in a differential mode operation at higher GHSV (up to ~3 125 000 ml g_{cat}^{-1} h^{-1}) and low CO conversions (≤30 %) to eliminate possible mass- and heat-transfer limitations. For each run at a specified reaction temperature, the CO conversions at 20 min, 40 min, and 60 min were averaged and used for calculations of the specific rate. The turnover frequency (TOF) was then calculated based on the specific rate and the dispersion, where the dispersion of SACs was assumed to be 100% and that of the Au/CeO_2 NP catalysts was calculated according to the equation D = 0.9/d where D represents the dispersion and d is the average diameter of Au NPs (in nm). We have added these details into the Methods section in the revised manuscript.

3. In Figure S3, the light-off curves are given for all three catalysts, but the effect of water is only shown for the Pt-CeO2-SAC. For completeness the effect of water should also be shown for the other two catalysts.

Response: We thank the reviewer's excellent suggestion. The effect of water on the other two Au NP catalysts is now presented as "Run 3-W" on Figure S3C and Figure S3D, respectively. To make it clearer to the readers, we have added a note in the legend of Figure S3 in the revised manuscript.

4. L42/43: "…catalysts because the interfacial perimeter sites usually serve as the dominant active sites." This is certainly not true in general. In fact, there are many cases, in which the boundary sites is not the most active.

Response: We thank the reviewer for identifying this problem in our original manuscript. We have replaced the word "usually" with "in many cases", thus the sentence has been changed into "*…catalysts because the interfacial perimeter sites **in many cases** serve as the dominant active sites.*"

5. L. 31/32: "…due to the quantum size and ensemble effects of metal nanoparticles…". Talking about heterogeneous catalysis in general, there are other effects that may play a role, for example the electronic and support effects discussed in this work.

Response: We appreciate the reviewer's comments. In order to better express our intention we have added the "and the differences between SACs and nanocatalysts in electronic and support effects as well as metal-support interaction" into the revision (*L.32/33* and *L.48/49*).

6. Figure 3: the arrow for CO2 has the wrong orientation (against the catalytic cycle).

Response: We thank the reviewer for kindly pointing this error. We have corrected the arrow in Figure 3 of the revised manuscript.

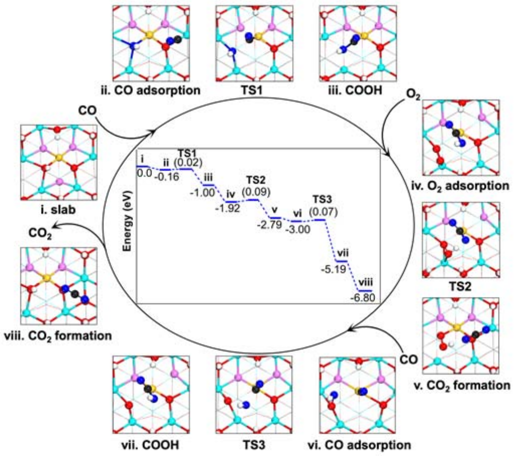

Figure R5 Reaction pathway B for CO oxidation on the Au_1/CeO_2 (111). The inset (within the ellipse) shows the potential energy profile and the numbers in the parentheses indicate the barriers of elementary steps.

7. Line 267: "which makes the reaction exothermic", change to "which makes the reaction step exothermic" (the overall thermodynamics is not changed).

Response: Thank you so much for kindly pointing this out. We have changed the sentence in the revised manuscript as "The presence of the highly positively charged Au_1 single atoms is crucial to the CO + OH reaction channel due to their flexibility in variation of oxidation state; the Au_1 atom can be reduced from Au(III) to Au(II) or Au(I), with Bader charge decreased from +1.10 to +0.69 eV during the CO oxidation reaction (Figure S17 and Table S2), which makes the reaction step exothermic.".

Reviewer #3 (Remarks to the Author):

This work analyses the catalytic performances of a promising catalyst Au1/CeO2, that was already proposed by a subset of authors (see Ref. 19, ACS Catalysis 2015), and that displays good performances towards CO oxidation as compared to commercial systems. In particular here the authors proposes that the fast deactivation of these Au/CeO2 catalysis originates from the consumption of OH species rather than from the sintering of the Au1 adatoms. In my opinion this is an interesting well-written article, that however presents a series of issues that I would ask the authors to address before I can give a recommendation on this work. On a more general level, I find that the main message, although interesting and deserving to be published on a good journal, could be better suited to a more specialised journal (i.e. catalysis).

1. The authors do not address at all the issue of sintering of Au species. I think that they should provide a more solid evidence that sintering can be excluded.

Response: We appreciate the reviewer's nice comments on our work and excellent questions that help us improve our manuscript. We acknowledge that the synthesis of Au_1/CeO_2 catalyst itself is not new and has been previously reported by us (*ACS Catal.* 2015, **5**, 6249-6254). However, the novelty of this work is not a new catalyst but the rather astonishing water promoting effect on Au_1/CeO_2 SAC compared with that on the Au/CeO_2 NP catalysts. Furthermore, our current work has provided deeper insights into why the Au_1 atoms and the perimeter Au atoms in supported Au NP catalysts are catalytically different. The extremely high activity of the Au_1/CeO_2 SAC under moisture conditions makes such catalysts more valuable for practical applications.

The referee raised a very important point about sintering of Au_1 SAC. In order to more clearly demonstrate that our Au_1/CeO_2 SAC did not sinter during the CO oxidation we have examined the used catalyst after deactivation at 200 ^oC. Figure R6 below clearly shows that after use the Au_1/CeO_2 SAC does not contain any Au clusters or NPs but only isolated Au atoms, which agrees with the theoretical results that the atoms are strongly bound with the surface oxygen atoms via covalent and ionic interactions.

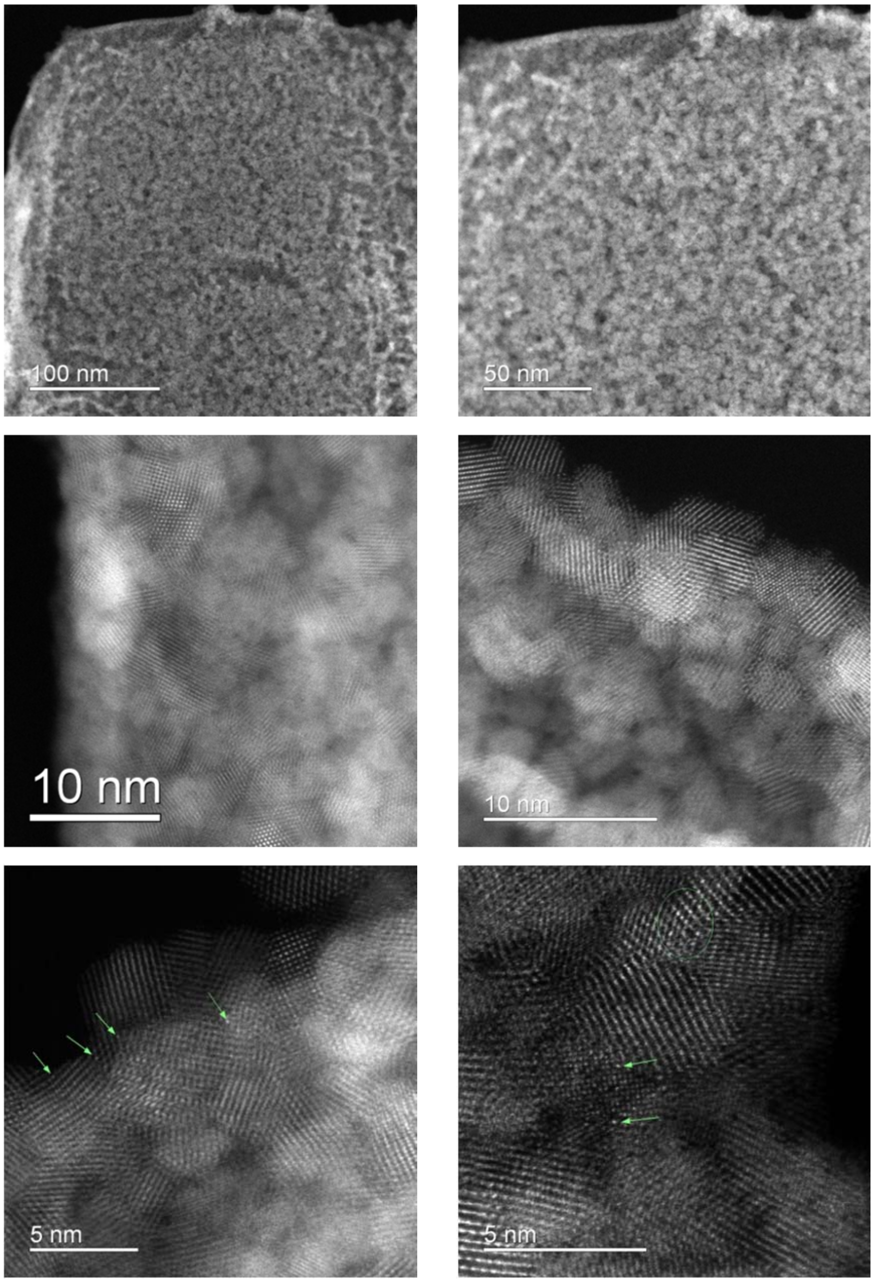

Figure R6 Representative HAADF-STEM images of the used Au_1/CeO_2 with different magnifications.

2. The nomenclature and charge of the Au1/CeO2 system is puzzling. As far as I understand the Au1 atom is substitutional for a surface Ce atom. The system is therefore more similar to a solid solution than to a supported catalyst. This could explain why sintering is not observed, although - again - the authors should clarify it (see point 1). What is puzzling is the charge of this Au1 atom, reported to be "positively charged, non-zero valentine's Au^{\delta +} atom. In the literature, this notation implicitly suggest a small positive charge. Instead, for a solid solution, the ionic nature of the Au1 species should be prominent, with oxidation states close to 3+ or 4+, as previously reported on the basis of

photoemission spectroscopy. This issue should in my opinion made clear, and some experimental data assessing the charge should be presented.

Response: We appreciate the reviewer's comments and suggestions. The referee is absolutely right. In our Au_1/CeO_2 SAC the Au single atoms are indeed located at the Ce vacancy sites (i.e. substitutional) as evidenced by ac-STEM-HAADF images (Figure S1 and images in our previous work (*ACS Catal.* 2015, **5**, 6249-6254)). In this case, the Au atoms should exist in a high **oxidation** state (e.g. Au(III) or Au^{+3}, which is often written as Au^{3+} **charge** states). Although oxidation state of Au can be measured by XPS or XANES, experimentally proving the Au oxidation state in our case is very challenge due to the extremely low loading of the Au atoms. In our previous report, we have examined the oxidation state of Au in Au_1/CeO_2 with an Au loading of 0.05 wt% (*ACS Catal.* 2015, **5**, 6249-6254). As shown in Figure R7, although the Au4f signal is very weak we can still deduce that the Au atom likely exists mainly as Au^{+} and Au^{3+} oxidation state, as is expected. Given the non-zero oxidation state (Au in Au-NPs are in zero oxidation state), the Au atoms carries positive charges, as confirmed by the calculated Bader atomic charge of Au_1^{δ} (δ = +1.26, see Table R1 below). Usually the effective atomic charge is only about 1/3 ~ 1/5 of the formal charge state or oxidation state (e.g. in PuO_2^{+}, Pu has oxidation state +V or charge state 5+, but the calculated atomic charge is only around +1.0|e^-|, as is discussed in our papers on this topic: Inorg. Chem. 2016 55,4616; Angew. Chem. Int. Ed. 2018, 57, 3242–3245). Because of this, we think the "positively charged, non-zero valent" feature of Au is reasonable.

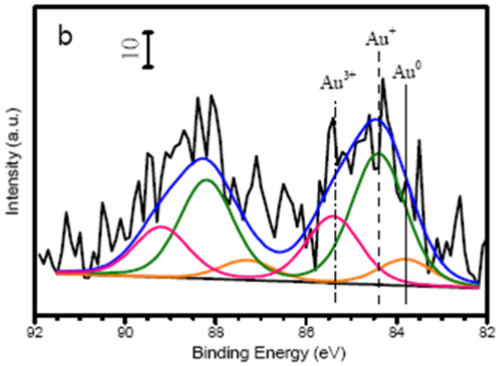

Figure R7 XPS of Au (4f) core level region in Au_1/CeO_2.

3. The computational study model the Au1/CeO2 system on the basis of a substitutional Au atom on a flat (111) surface. The authors should provide more solid evidence that this highly ideal model does capture the physical features of the new catalyst. For example, the Au atom in this model will be highly ionic (see point 2).

Response: This is a good suggestion. Indeed, as shown experimentally Au_1 substitutes the Ce atom in

ceria, thus existing in a positive oxidation state. To estimate the possible oxidation states of Au atoms in the catalysts, we have now calculated the Bader charges of Au atoms in the Au_1/CeO_2 as well as in the $Au(OH)_x$ (x = 1-3) molecules as a comparison. As shown in Table R1, Au atom in the Au_1/CeO_2-OH is indeed positively charged (atomic charge +1.10 $|e^-|$) and the calculated atomic charge is close to that in $Au(OH)_3$ (+0.988 $|e^-|$), indicating that the oxidation state of Au_1 can be assigned as Au(III) and the charge state is thus Au^{3+}, which is consistent with previous studies [*Nano Res*. 2015, 8(9): 2913-2924]. As presented in the projected DOS, Au 5d-orbitals indeed have two peaks above the Fermi level, suggesting the formation of an Au^{3+} cation. The additional results also confirm the high oxidation states and positively charged Au atoms in Au_1/CeO_2 SAC. We have added the new data into Table S2 and Figure S17 in the revised support information.

Table R1 Calculated oxygen vacancy (O_v) formation energies and Bader atomic charges of Au in Au_1 and Au_{13} nanoparticle with different models, and the Bader charges of $Au(OH)_x$ (x = 1-3) molecules listed for comparison.

	models	Charge of Au $\lvert e^-\rvert$	Reaction heat for CO+OH → COOH (eV)	Charge of Au after CO+OH →COOH reaction $\lvert e^-\rvert$	Oxygen vacancy formation energy (eV)
Au_1/CeO_2	A	+1.26	--	--	0.13
Au_1/CeO_2-OH	B	+1.10	-0.84	+0.69	1.42
Au_1/CeO_2-3O	C	--	--	--	1.23
Au_{13}/CeO_2-OH	A/B	+0.01	0.67	+0.02	2.26
Au_{13}/CeO_2	C	+0.03	-	--	2.29
Au-OH		+0.372			
$Au-(OH)_2$		+0.690			
$Au-(OH)_3$		+0.988			

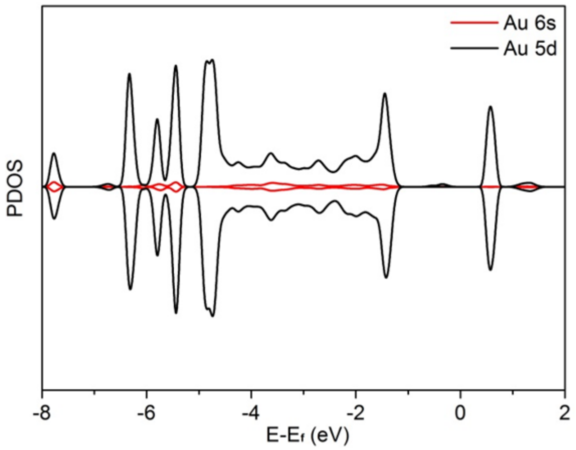

Figure R8 Projected electronic density of state (PDOS) for Au atom in Au_1/CeO_2-OH model. Energies are referred to the Fermi level (ε_F =0).

REVIEWERS' COMMENTS:

Reviewer #1 (Remarks to the Author):

The manuscript was previously revised and some questions were addressed to the authors. In the response, the authors clarify well all the doubts raised, so in my opinion, this work can be published with the modifications made.

Reviewer #2 (Remarks to the Author):

The authors have addressed the remaining (minor) comments convincingly. I recommend this very nice piece of work for publication in Nature Communications.

Response to reviewers’ comments

Reviewer #1 (Remarks to the Author):

The manuscript was previously revised and some questions were addressed to the authors. In the response, the authors clarify well all the doubts raised, so in my opinion, this work can be published with the modifications made.

Answer： Thank you very much for your nice comments!

Reviewer #2 (Remarks to the Author):

The authors have addressed the remaining (minor) comments convincingly. I recommend this very nice piece of work for publication in Nature Communications.

Answer： Thank you very much for your nice comments!
